# A Novel Framework for Characterizing Genomic Haplotype Diversity in the Human Immunoglobulin Heavy Chain Locus

**DOI:** 10.3389/fimmu.2020.02136

**Published:** 2020-09-23

**Authors:** Oscar L. Rodriguez, William S. Gibson, Tom Parks, Matthew Emery, James Powell, Maya Strahl, Gintaras Deikus, Kathryn Auckland, Evan E. Eichler, Wayne A. Marasco, Robert Sebra, Andrew J. Sharp, Melissa L. Smith, Ali Bashir, Corey T. Watson

**Affiliations:** ^1^Department of Genetics and Genomic Sciences, Icahn School of Medicine at Mount Sinai, New York, NY, United States; ^2^Department of Biochemistry and Molecular Genetics, University of Louisville School of Medicine, Louisville, KY, United States; ^3^Wellcome Centre for Human Genetics, University of Oxford, Oxford, United Kingdom; ^4^Department of Genome Sciences, University of Washington School of Medicine, Seattle, WA, United States; ^5^Howard Hughes Medical Institute, University of Washington, Seattle, WA, United States; ^6^Department of Cancer Immunology and AIDS, Dana-Farber Cancer Institute, Department of Medicine, Harvard Medical School, Boston, MA, United States; ^7^Icahn Institute of Data Science and Genomic Technology, New York, NY, United States

**Keywords:** immunoglobulin heavy chain locus, single nucleotide variation, structural variation, antibody, B cell receptor, long-read sequencing

## Abstract

An incomplete ascertainment of genetic variation within the highly polymorphic immunoglobulin heavy chain locus (IGH) has hindered our ability to define genetic factors that influence antibody-mediated processes. Due to locus complexity, standard high-throughput approaches have failed to accurately and comprehensively capture IGH polymorphism. As a result, the locus has only been fully characterized two times, severely limiting our knowledge of human IGH diversity. Here, we combine targeted long-read sequencing with a novel bioinformatics tool, IGenotyper, to fully characterize IGH variation in a haplotype-specific manner. We apply this approach to eight human samples, including a haploid cell line and two mother-father-child trios, and demonstrate the ability to generate high-quality assemblies (>98% complete and >99% accurate), genotypes, and gene annotations, identifying 2 novel structural variants and 15 novel IGH alleles. We show multiplexing allows for scaling of the approach without impacting data quality, and that our genotype call sets are more accurate than short-read (>35% increase in true positives and >97% decrease in false-positives) and array/imputation-based datasets. This framework establishes a desperately needed foundation for leveraging IG genomic data to study population-level variation in antibody-mediated immunity, critical for bettering our understanding of disease risk, and responses to vaccines and therapeutics.

## Introduction

Defining the factors that contribute to differences in the antibody (Ab) response is critical to furthering our understanding of immunological diseases, and informing the design of vaccines and therapeutics. Antibodies are an extremely diverse protein family in humans, encoded by >100 highly homologous gene segments that reside within one of the most structurally complex and polymorphic regions of the human genome (1–3). The immunoglobulin heavy chain (IGH) locus, specifically, consists of >50 variable (IGHV), >20 diversity (IGHD), 6 joining (IGHJ), and 9 constant (IGHC) functional/open reading frame (F/ORF) genes that encode the heavy chains of expressed Abs (4). Greater than 250 IGH gene segment alleles are curated in the ImMunoGeneTics Information System (IMGT) database (4), and this number continues to increase (2, 5–11). The locus is enriched for single nucleotide variants (SNVs) and large structural variants (SVs) involving functional genes (5, 12–19), at which allele frequencies are known to vary among human populations (2, 19, 20). The formation of the Ab repertoire is mediated by several complex molecular processes and can be influenced by many factors. Studies in twins have demonstrated that features of the Ab repertoire are heritable, and IG germline variants have been shown to directly impact antibody usage and antigen-specificity (20–22). Together this highlights the need to better understand the genetic factors that contribute to variation in antibody-mediated immunity.

Currently, existing genomic resources and tools for the IG loci are incomplete and poorly represent germline diversity across human populations. Historically, the complexity of the IGH locus has hindered our ability to comprehensively characterize polymorphisms within this region using high-throughput approaches (3, 23). In fact, only two complete haplotypes in IGH have been fully resolved and characterized (1, 2). As a result, IGH has been largely overlooked by genome-wide studies, leaving our understanding of the contribution of polymorphisms within IGH in antibody-mediated immunity incomplete (2, 3, 23). While early studies uncovered associations to disease susceptibility within IGH, few links have been made by genome-wide association studies (GWAS) and whole genome sequencing (WGS) (3, 24, 25). Moreover, little is known about the genetic regulation of the human Ab response.

To define the role of IGH variation in Ab function and disease, all classes of variation must be resolved (5, 13, 16, 20, 26, 27). Although approaches have been developed for utilizing genomic or Adaptive Immune Receptor Repertoire sequencing (AIRR-seq) data, variant calling and broad-scale haplotype inference are restricted primarily to coding regions (7, 8, 17–19, 28). To fully characterize genetic diversity in the IG loci, specialized genotyping methods capable of capturing locus-wide polymorphism with nucleotide resolution are required. Indeed, such methods have been applied to better resolve complex and hyper-polymorphic immune loci elsewhere in the genome (29, 30).

Long-read sequencing technologies have been used to detect chromosomal rearrangements (31), novel SVs (32, 33), and SVs missed by standard short-read sequencing methods (34, 35), including applications in the complex killer immunoglobulin-like receptors (KIR) (36) and human leukocyte antigen (HLA) (31, 37, 38) loci. Furthermore, the sensitivity of SV detection is improved by resolving variants in a haplotype-specific manner (35, 39). When long-read sequencing has been combined with specific target enrichment methods, using either a CRISPR/Cas9 system (40, 41) or DNA probes (42, 43), it has been shown to yield accurate and contiguous assemblies. Targeted approaches have enabled higher resolution genotyping of the HLA loci (44) and KIR regions (45, 46).

**FIGURE 1:**
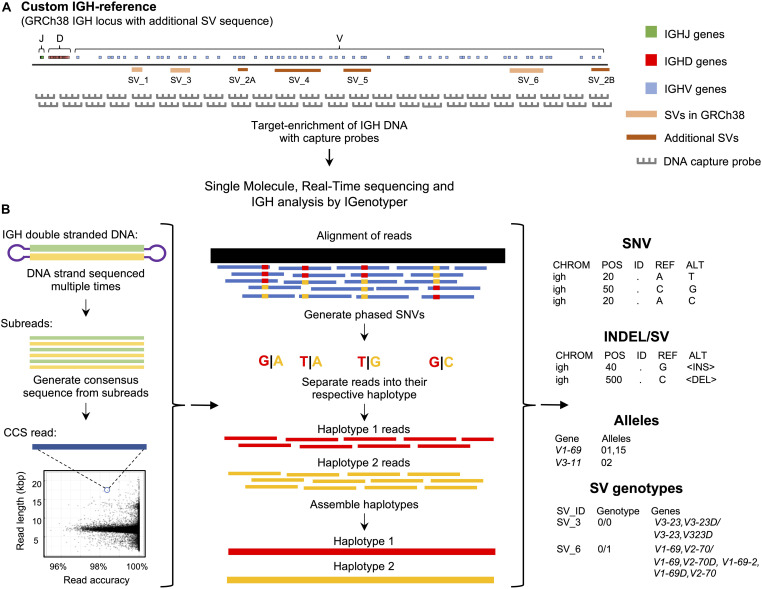
An overview of the custom capture and IGenotyper workflow used to detect IGH variation in a haplotype-specific manner. **(A)** A schematic of the custom IGH-reference used in this study, which includes the GRCh38 IGH locus with the addition of known SV sequences inserted into their respective positions in the locus. Brown bars indicate the positions of inserted SVs in the IGH-reference; pink bars indicate the positions of additional known SVs present in GRCh38 relative to GRCh37. Positions of IGHJ (green), IGHD (red) and IGHV (blue) genes in the IGH-reference are also indicated. The oligo capture probes used in the panels designed for this study were based on the sequence of this custom IGH-reference. **(B)** After targeted long-read capture, constructed libraries undergo SMRT sequencing, and the resulting data is processed with IGenotyper. Raw SMRT sequences with at least 2 subreads are converted into higher accuracy CCS reads. Phased SNVs are identified from the CCS reads and used to phase both the CCS reads and subreads, which are then used to assemble haplotype-specific assemblies of IGH. SNVs, indels, SVs and IG gene/alleles are identified from the assembly and CCS reads. SVs embedded into the IGH-reference are genotyped using the assembly, CCS reads, and SNV calls.

Here, we present a novel framework that utilizes IGH-targeted long-read sequencing, paired with a new IG genomics analysis tool, IGenotyper^1^, to characterize variation in the IGH locus (Figure 1). We apply this strategy to eight human samples, and leverage orthogonal data and pedigree information for benchmarking and validation. We demonstrate that our approach leads to hiqh-quality assemblies across the IGH locus, allowing for comprehensive genotyping of SNVs, insertions and deletions (indels), SVs, as well as annotation of IG gene segments, alleles, and associated non-coding elements. We show that genotype call sets from our pipeline are more comprehensive than those generated using alternative short-read and array-/imputation-based methods. Finally, we demonstrate that use of long-range phasing/haplotype information improves assembly contiguity, and that sample multiplexing can be employed to scale the approach in a cost-effective manner without impacting data quality.

## Materials and Methods

### Library Preparation and Sequencing

Genomic DNA samples were procured from Coriell Repositories (1000 Genomes Project, 1KGP donors; Camden, NJ, United States) and collaborators. Briefly, 1–2 micrograms of high molecular weight genomic DNA from each sample was sheared using g-tube to ∼8 Kb (Covaris, Woburn, MA, United States). These sheared gDNA samples were size selected to include 5–9 Kb fragments using a BluePippin (Sage Science, Beverly, MA, United States). Following size selection, each sample was End Repaired and A-tailed following the standard KAPA library preparation protocol (Roche, Basel, Switzerland). For multiplexed samples, adapters containing sequence barcodes (Pacific Biosciences, Menlo Park, CA, United States) and a universal priming sequence were ligated onto each sample. Each sample was PCR amplified for 9 cycles using HS LA Taq (Takara, Mountain View, CA, United States) and cleaned with 0.7X AMPure beads to remove small fragments and excess reagents (Beckman Coulter, Brea, CA, United States). The genomic DNA libraries were then captured following the SeqCap protocol which was modified to increase final capture reaction volume by 1.5X (Roche, Basel, Switzerland). Following capture, the libraries were washed following the SeqCap protocol, substituting vortexing with gentle flicking. The washed capture libraries were PCR amplified for 18 cycles using HS LA Taq and cleaned with 0.7X AMPure beads.

Capture libraries were prepared for PacBio sequencing using the SMRTbell Template Preparation Kit 1.0 (Pacific Biosciences, Menlo Park, CA, United States). Briefly, each sample was treated with a DNA Damage Repair and End Repair mix in order to repair nicked DNA. SMRTbell adapters were ligated onto each capture library to complete SMRTbell construction. The SMRTbell libraries were then treated with exonuclease III and VII to remove any unligated gDNA and cleaned with 0.45X AMPure PB beads (Pacific Biosciences, Menlo Park, CA, United States). Resulting libraries were prepared for sequencing according to the manufacturer’s protocol and sequenced as single libraries per SMRTcell with P6/C4 chemistry and 6 h movies on the RSII system, or as multiplexed libraries per SMRTcell 1M, annealed to primer V4 and sequenced using 3.0 chemistry and 20 h movies, on the Sequel system (see Supplementary Table S4 for details).

### Creating a Custom IGH Locus Reference

The IGH locus, excluding the IGH constant gene region (chr14:106,326,710-107,349,540) was removed from the human genome reference build GRCh37, and the expanded custom IGH locus reference was inserted in its place. The expanded custom IGH locus includes sequence spanning IGH from the GRCh38 reference assembly, plus the addition of known SVs. The included SVs were previously characterized from fosmid clones AC244473.3, AC241995, AC234225, AC233755, KC162926, KC162924, AC231260, AC244456 and KC162925, and sequence from human genome reference build GRCh37 (chr14:106,527,905-106,568,151). The IGenotyper toolkit command ‘IG-make-ref’ takes as input the human genome reference build GRCh37 and creates the custom IGH locus reference.

### IGenotyper: A Streamlined Analysis Tool for IGH Locus Assembly, Variant Detection/Genotyping, and Gene Feature Annotation

Running IGenotyper returns multiple output files: (1) the alignment of the circular consensus sequence (CCS) reads and assembled locus to the reference in BAM format; (2) the assembled IGH locus in FASTA format, (3) the SNVs in VCF; (4) indels and SVs in BED format; (5) a parsable file with genotyped SVs; (6) a parsable file with the detected alleles for each functional/ORF gene; and (7) several tab delimited files detailing different sequencing run and assembly statistics. The BAM file contains phased CCS reads and includes haplotype annotation in the read group tag of every read. This allows the user to separate the reads into their respective haplotype in the Integrative Genomics Viewer (IGV) visualization tool (47). The VCF file contains annotations indicating whether SNVs reside within SV regions, and IG gene features, including coding, intron, leader part 1 (LP1), and recombination signal (RS) sequences. A user-friendly summary file is produced with links to output files (see Supplementary Figure S1; sample summary output for NA19240), including summary tables and figures pertaining to: locus sequence coverage; counts of SNVs, indels and SVs; allele annotations/genotypes for each IGHV, IGHD, and IGHJ genes; and lists of novel alleles.

#### Running IGenotyper

IGenotyper has three main commands: ‘phase‘, ‘assemble‘, and ‘detect‘. The input is the subread bam output from the RSII or Sequel sequencing run. ‘phase‘ phases the subreads and CCS reads. ‘assemble‘ partitions the IGH locus into haplotype-specific regions and assembles each region. ‘detect‘ detects SNVs, indels and SVs, genotypes 5 SVs embedded in the IGH reference and assigns the IGH genes to alleles from the IGMT database.

#### Phasing SMRT Sequencing Reads

Raw SMRT sequences with at least 2 subreads are converted into consensus sequences using the ‘ccs‘ command^2^. CCS reads and subreads are aligned to our expanded custom IGH locus reference using BLASR (48). Phased SNVs are detected from the CCS reads using the WhatsHap (49) ‘find_snv_candidates‘, ‘genotype‘, and ‘phase‘ commands. The subreads and CCS reads are phased using the command ‘phase-bam‘ from MsPAC (50).

#### Assembling the IGH Locus

Haplotype blocks are defined using the WhatsHap ‘stats‘ command. Haplotype-specific CCS reads within each haplotype block are assembled separately using Canu (51); regions outside haplotype blocks are assembled using all aligned CCS reads. Regions lacking contigs recruit raw subreads and repeat the assembly process. Contigs with a quality score less than 20 are filtered.

#### Detecting Variants From SNVs to SVs

SNVs are detected from the assembly aligned to the reference. SNVs in the VCF are annotated with:

1. Contig id used to detect the SNV.
2. Overlapping SV id (if any).
3. A true or false value if the SNV is also detected by the CCS reads.
4. Whether the SNV falls within an intron, leader part 1 sequence or gene exon.
5. Whether the SNV is within the IGHV, IGHD, or IGHJ region.
6. For phased blocks, the haplotype block id and genotype.

Indels and SVs are detected using the ‘sv-calling‘ command from MsPAC (50). Importantly, each indel and SV is sequence-resolved since they are identified from a multiple sequence alignment using the haplotype-specific assemblies and reference.

#### Assigning Alleles to IGH Genes Extracted From the Assembly

Assembled sequences overlapping the IGH genes are extracted and compared to the alleles in the IMGT database (v202031-2). Sequences not observed in the database are labeled as novel. CCS reads overlapping IGH gene sequences are also extracted and compared to the IMGT database. To provide supporting evidence for the allele, a count for the number of CCS reads with the same sequence found in the assembly is reported (currently only supported for Sequel data). Assembly and CCS sequences are outputted in a FASTA file; gene names, alleles (and allele sequence if novel), and read support are outputted in a tab-delimited file.

### Validating IGenotyper Assemblies

#### Assessing the Accuracy of the NA12878 and NA19240 Assemblies

The accuracy of assemblies for NA19240 and NA12878 was assessed by BLAST (v2.7.1+) alignment of contigs to fosmids assembled from Sanger sequencing (NA19240 accession numbers: AC241513.1, AC234301.2, KC162926.1, AC233755.2, AC234135.3, AC244463.2; NA12878 accession numbers: AC245090.1, AC244490.2).

The accuracy of the assemblies was also assessed using Illumina data. Illumina HiSeq 2500 2 × 126 paired-end, PCR-free sequencing data (ERR894723, ERR894724, ERR899709, ERR899710 and ERR899711) was downloaded from the European Nucleotide Archive and aligned to the NA19240 assembly using bwa mem (v0.7.15-r1140). The total coverage across all sequencing runs was 75.6x and Pilon (v1.23) reported an accuracy of 99.996 (102 errors in 2,396,307 bp). Additionally, Illumina NovaSeq 6000 2 × 151 paired-end, TruSeq PCR-free sequencing run ERR3239334 was downloaded from the European Nucleotide Archive and aligned to the NA12878 assembly. The coverage was 35x and Pilon reported an accuracy of 99.991 (167 errors in 1,918,794 bp).

### Manual Curation of IGHV3-30 and IGHV1-69 Gene Regions

*IGHV3-30* and *IGHV1-69* gene duplication regions did not completely assemble into a single contig per haplotype, but instead were split into multiple contigs. To resolve these regions an additional curation step was employed: contigs were aligned to each other using BLAST and overlapping contigs with high alignment score were merged.

In NA19240, the *IGHV3-30* gene region duplication was initially assembled into 8 contigs. Two contigs were merged to form a novel SV containing a ∼25 Kb deletion relative to the IGH-reference. The two contigs overlapped by 7,706 bp with 5 bp mismatches and 9 gaps (11 gap bases). The alternate haplotype was initially assembled into 6 contigs. The 6 contigs overlapped by more than 2.3 Kbp with 0 bp mismatches and a total of 8 gap bases, allowing them to be merged into a single contig. Both haplotypes were validated with fosmids and assemblies from the parents. The resulting contigs resolved the SVs on both haplotypes. This process was repeated for NA12878, and in both probands for the *IGHV1-69* gene region.

Leveraging parental and fosmid assembly data, we determined that NA19240 carried three distinct haplotypes within the SV region spanning *IGHV1-69*, *IGHV2-70D*, *IGHV1-69-2*, *IGHV1-69D*, and *IGHV2-70*. An insertion haplotype carrying all genes within the region was paternally inherited; a deletion haplotype, lacking *IGHV2-70D*, *IGHV1-69-2*, and *IGHV1-69D*, was inherited from the mother; and a second deletion haplotype was detected in both the capture/IGenotyper and fosmid assembly data, but was not supported by either parental dataset. This deletion haplotype was identical to the paternally derived insertion haplotype on the flanks of the deletion event, suggesting it represented a somatic SV. Whether this arose natively in NA19240 or is an artifact found only within the LCL is not known. To construct the most accurate assemblies of the inherited haplotypes, we attempted to remove reads representing this somatic deletion and performed a local reassembly. This allowed us to produce more accurate contigs across this region which exhibited higher concordance to both fosmid and parental datasets.

### Comparing Variants From IGenotyper to Other Datasets

#### Comparing Illumina and IGenotyper SNV Calls in CHM1

SNVs from our assembly and Illumina reference alignment to GRCh37 were compared to a ground truth SNV dataset generated by aligning GRCh38 region chr14:106,329,408-107,288,965 to GRCh37 using pbmm2 (v1.0.0). Positions with base differences in the alignment were labeled as SNVs. 2 × 126 TruSeq PCR-free Illumina libraries (SRR3099549 and SRR2842672) were aligned to GRCh37 with bwa (0.7.15-r1140) and SNVs were detected using the standard protocol with GATK (v3.6) tools, HaplotypeCaller and GenotypeGVCFs (52). The SNVs were filtered using bcftools (v1.9) for SNVs with genotype quality greater than 60 and read depth greater than 10.

#### Comparing 1000 Genomes Project SNVs to NA12878 and NA19240 SNVs Detected by IGenotyper

SNVs detected by IGenotyper were compared to SNVs from the 1KGP phase 3 dataset. IGH-specific SNVs from the 1KGP (ftp://ftp.1000genomes.ebi.ac.uk/vol1/ftp/release/2013 0502/supporting/hd_genotype_chip/ALL.chip.omni_broad_sang er_combined.20140818.snps.genotypes.vcf.gz, ftp://ftp.1000 genomes.ebi.ac.uk/vol1/ftp/release/20130502/ALL.chr14.phase3_ shapeit2_mvncall_integrated_v5a.20130502.genotypes.vcf.gz,ftp: //ftp.1000genomes.ebi.ac.uk/vol1/ftp/release/20130502/support ing/hd_genotype_chip/ALL.chip.omni_broad_sanger_combined. 20140818.snps.genotypes.vcf.gz, and ftp://ftp.1000genomes.ebi. ac.uk/vol1/ftp/phase3/data/NA19240/cg_data/NA19240_lcl_SRR 832874.wgs.COMPLETE_GENOMICS.20130401.snps_indels_sv s_meis.high_coverage.genotypes.vcf.gz) were extracted using ‘bcftools view –output-type v, –regions 14:106405609-107349540, –min-ac 1, –types snps‘. Overlap between the 1KGP Phase 3 SNVs and SNVs detected by IGenotyper was determined using BEDTools ‘intersect‘ command. Overlapping SNVs with discordant genotypes were labeled as discordant/non-overlapping SNVs.

#### Comparing Indels, SVs, and BioNano Data From the 1000 Genome Structural Variation Consortium

Indels and SVs detected by IGenotyper were compared to indels and SVs from the Human Genome Structural Variation (HGSV) Consortium (ftp://ftp.1000genomes.ebi.ac.uk/vol1/ftp/data_collections/hgsv_sv_discovery/working/integration/201 70515_Integrated_indels_Illumina_PacBio/Illumina_Indels_Mer ged_20170515.vcf.gz, ftp://ftp.1000genomes.ebi.ac.uk/vol1/ftp/data_collections/hgsv_sv_discovery/working/20180627_Phased SVMSPAC/PhasedSVMsPAC.NA19240.vcf). Additionally, BioNano SV calls (ftp://ftp.1000genomes.ebi.ac.uk/vol1/ftp/data_collections/hgsv_sv_discovery/working/20180502_bionano/GM19240_DLE1_SV_hg38_indel.vcf and ftp://ftp.1000 genomes.ebi.ac.uk/vol1/ftp/data_collections/hgsv_sv_discovery/working/20170109_BioNano_SV_update/GM19240_YRI_Daug hter_20170109_bionano_SVMerged_InDel.vcf.gz) were used to validate SVs identified by IGenotyper in NA19240.

#### Analysis of SNVs in Regions Accessible to Next Generation Sequencing Methods

SNVs were evaluated to determine if they were within regions accessible by next generation sequencing methods. The “strict” accessibility track was converted to bed format from:

https://hgdownload.soe.ucsc.edu/gbdb/hg19/1000Genomes/phase3/20141020.strict_mask.whole_genome.bb.

The number of SNVs within the “strict” accessible regions was determined by using the BEDtools “intersect” command.

#### Calculating Hardy-Weinberg Equilibrium at NA19240 and NA12878 SNVs

SNVs from the 1KGP (phase 3) were downloaded from ftp:// ftp.1000genomes.ebi.ac.uk/vol1/ftp/release/20130502/ALL.chr14. phase3_shapeit2_mvncall_integrated_v5a.20130502.genotypes.v cf.gz. Samples corresponding to the “EUR” superpopulation were extracted from the VCF file, and samples corresponding to the “AFR” superpopulation were extracted into a separate VCF file. Hardy-Weinberg Equilibrium (HWE) was calculated using vcftools with the option “—hardy.”

## Results

### Novel Tools for Comprehensively Characterizing IG Haplotype Diversity

To interrogate locus-wide IGH variants, we implemented a framework that pairs targeted DNA capture with single molecule, real time (SMRT) sequencing (Pacific Biosciences) (Figure 1A). Roche Nimblegen SeqCap EZ target-enrichment panels (Wilmington, MA, United States) were designed using DNA target sequences from the human IGH locus. Critically, rather than using only a single representative IGH haplotype (e.g., those available as part of either the GRCh37 or GRCh38 human reference assembly) we based our design on non-redundant sequences from the GRCh38 haplotype (2), as well as additional complex SV and insertion haplotypes distinct from GRCh38 (1, 2) (Figure 1A and Supplementary Table S1). Additional details, including the exact target sequences used and additional specifications of these capture panels are provided in the Supplementary Material (Supplementary Note 1 and Tables S2, S3).

To process and analyze these long-read IGH genomic sequencing data, we developed IGenotyper (Figure 1B)^3^ which utilizes and builds on existing tools to generate diploid assemblies across the IGHV, IGHD, and IGHJ regions (see section “Materials and Methods”); for ease, we refer to these three regions (excluding IGHC) collectively as IGH. From generated assemblies, IGenotyper additionally produces comprehensive summary reports of SNV, indel, and SV genotype call sets, as well as IG gene/allele annotations. For read mapping, SNV/indel/SV calling, and sequence annotation, the pipeline leverages a custom IGH locus reference that represents known SV sequences in a contiguous, non-redundant fashion (Figure 1A); this reference harbors the same sequence targets used for the design of target-enrichment panels, and ensures that known IGH SVs in the human population can be interrogated.

### Benchmarking Performance Using a Haploid DNA Sample

We first benchmarked our performance using genomic DNA from a complete haploid hydatidiform mole sample (CHM1), from which IGH had been previously assembled using Bacterial Artificial Chromosome (BAC) clones and Sanger sequencing (2). This reference serves as the representation of IGH in GRCh38. Using panel designs mentioned above, we prepared two SMRTbell libraries with an average insert size of 6–7.5 Kb for sequencing on both the RSII and Sequel systems (Supplementary Table S4). We observed a mean subread coverage across our custom IGH reference (Figure 1A) of 557.9x (RSII) and 12,006.4x (Sequel 1), and mean circular consensus sequence (CCS) read coverage of 45.1x (RSII) and 778.2x (Sequel 1). The average Sequel CCS phred quality score was 70 (99.999991% accuracy), with an average read length of 6,457 bp (Supplementary Figure S2). Noted differences in target-enrichment panels tested are described in Supplementary Note 1.

**FIGURE 2:**
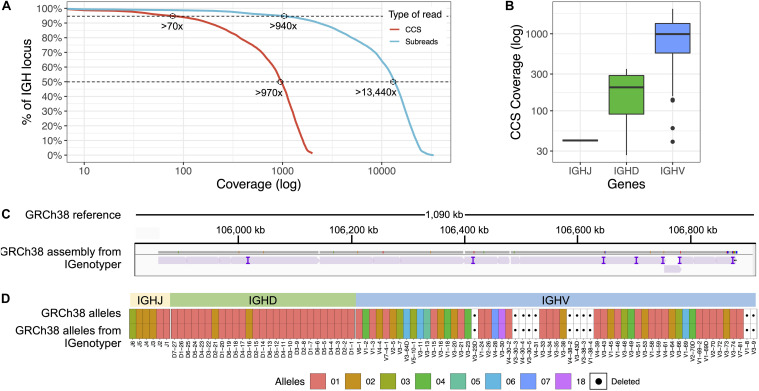
Benchmarking targeted long-read sequencing and assembly in a haploid DNA sample. **(A)** The empirical cumulative subread (blue) and CCS (red) coverage in IGH from the combined CHM1 dataset. The subread coverage for 95 and 50% (dotted line) of the locus is greater than 940× and 13,440×, and the CCS read coverage for 95 and 50% of the locus is greater than 70× and 970×, respectively. **(B)** CCS coverage across IGHJ, IGHD and IGHV genes. The average CCS coverage of IGHV genes was >1,000×. **(C)** IGenotyper assembly of CHM1 aligned to GRCh38. Purple tick marks represent indels in the IGenotyper assembly relative to GRCh38. **(D)** IGHJ, IGHD, and IGHV alleles detected by IGenotyper in CHM1 compared to alleles previously annotated in GRCh38.

To most effectively use these data to assess IGenotyper performance, we combined reads from both libraries for assembly (Supplementary Table S4). A total of 970,302 bp (94.8%) of IGH (chr14:105,859,947-106,883,171; GRCh38) was spanned by >1,000× subread coverage, and 1,006,287 bp (98.3%) was spanned by >20× CCS coverage (Figure 2A). The mean CCS coverage spanning IGHV, IGHD, and IGHJ coding sequences was 160.3× (median = 42.5×; Figure 2B). Compared to GRCh38, IGenotyper assembled 1,009,792 bases (98.7%) of the IGH locus in the CHM1 dataset (Figure 2C and Table 1). Gap sizes between contigs ranged from 177 to 3,787 bp (median = 456 bp) in length. Only 37 (<0.004% of bases) single nucleotide differences were observed when compared to GRCh38 (base pair concordance >99.99%). In addition, 220 potential indel errors were identified (Supplementary Table S5). The majority of these (199/220) were 1–2 bp in length, 61.8% of which (123/199) occurred in homopolymer sequences, consistent with known sources of sequencing error in SMRT sequencing and other technologies (Supplementary Table S6). We also observed a 2,226 bp indel consisting of a 59mer tandem repeat motif near the gene *IGHV1-69* (Supplementary Figure S3). This tandem repeat was unresolved in GRCh38 (Supplementary Figure S4 and Note 2), which was reconstructed using a Sanger shot-gun assembly approach (2); it remains unclear whether this event represents an improvement in the IGenotyper assembly over GRCh38, or is a sequencing/assembly artifact. Nonetheless, the total number of discordant bases associated with indels (2,521 bp) accounts for only <0.28% of the assembly.

**TABLE 1:** Assembly statistics and evaluation of the accuracy of the haplotype-specific assemblies.

**Sample**	**Contigs (n)**	**Assembly size (bp)**	**Assembly validation**		
			**Concordance with fosmids (SMRT sequencing)**	**Concordance with BACs or fosmids (Sanger sequencing)**	**Concordance with Pilon/Illumina**
CHM1	16	1,026,385	NA	99.996%	NA
NA19240	38	1,829,616	99.996%	99.99%	99.99%
NA12878	45	1,442,310	99.995%	100.0%	99.99%

All known SVs previously described in CHM1 (2) were present in the IGenotyper assembly, accounting for all IGHV (*n* = 47), IGHD (*n* = 27), and IGHJ (*n* = 6) F/ORF gene segments in this sample. In addition to genes previously characterized by BAC sequencing, the IGenotyper assembly additionally spanned *IGHV7-81*. Alleles identified by IGenotyper were 100% concordant with those identified previously in GRCh38 (Figure 2D) (2).

### Assessing the Accuracy of Diploid IGH Assemblies

We next assessed the ability of IGenotyper to resolve diploid assemblies in IGH, using a Yoruban (YRI; NA19240, NA19238, NA1239) and European (CEU; NA12878, NA12891, NA12892) trio from the 1000 Genomes Project [1KGP (53); Supplementary Figure S4 and Supplementary Table S4]. Lymphoblastoid cell lines (LCLs), which are the primary source of 1KGP sample DNA are known to harbor V(D)J somatic rearrangements within the IG loci, including reduced coverage in IGHD, IGHJ, and proximal IGHV regions (2, 19). However, because IGenotyper assembles the IGH locus in a local haplotype-specific manner, V(D)J rearrangements can be easily detected (Supplementary Figures S5,S6 and Table S7). Nonetheless, we focused our analysis exclusively on the IGHV region (9 Kb downstream of *IGHV6-1* to telomere) to avoid potential technical artifacts that would hinder our benchmarking assessment.

IGH enrichment was performed and libraries were sequenced on the RSII or Sequel platform (Supplementary Table S4). For diploid samples, IGenotyper (Figure 1B) first identifies haplotype blocks using CCS reads that span multiple heterozygous SNVs within a sample. Within each haplotype block, CCS reads are then partitioned into their respective haplotype and assembled independently to derive assembly contigs representing each haplotype in that individual. Reads within blocks of homozygosity that cannot be phased are collectively assembled, as these blocks are considered to represent either: (1) homozygous regions, in which both haplotypes in the individual are presumed to be identical, or (2) hemizygous regions, in which the individual is presumed to harbor an insertion or deletion on only one chromosome (Supplementary Figure S7).

**FIGURE 3:**
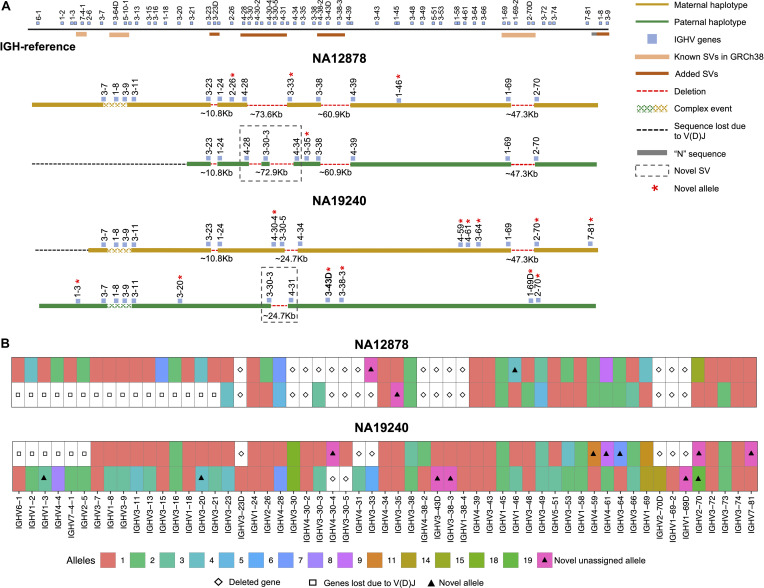
Haplotype-resolved assembly for characterizing structural variants and IGHV gene alleles in NA12878 and NA19240. **(A)** A schematic of the custom IGH-reference spanning the IGHV gene region (top). Brown bars indicate the positions of inserted SVs in the IGH-reference; pink bars indicate the positions of additional known SVs present in GRCh38 relative to GRCh37. Positions of IGHV (blue) genes are also indicated. Schematic depictions of resolved maternally (gold) and paternally (green) inherited haplotypes in NA12878 and NA19240 are shown. Detected deletions within annotated SV regions are labeled with a red dotted line, with detected sizes of each event also provided. Genes directly flanking or within detected SVs are labeled. Genes with novel alleles are labeled with red asterisks, and novel SVs are indicated with dotted boxes. **(B)** Alleles predicted by IGenotyper for each gene across both haplotypes in NA12878 and NA19240. Novel alleles are marked by a filled triangle, and deleted genes and genes not present due to V(D)J recombination are marked by a diamond and square, respectively.

We assessed IGenotyper performance in the probands of each trio. IGenotyper assemblies were composed of 41 and 49 haplotype blocks in NA19240 and NA12878, respectively (Supplementary Table S8). Of these, 20/41 and 24/49 blocks in each respective sample were identified as heterozygous, in which haplotype-specific assemblies could be generated, totaling 826,548 bp (69.28%) in NA19240, and 424,834 bp (35.61%) in NA12878. Within these heterozygous blocks, the mean number of heterozygous positions was 76.16 (NA19240) and 52.08 (NA12878). Summing the bases assembled across both heterozygous and homozygous/hemizygous contigs, complete assemblies comprised 1.8 Mb of diploid resolved sequence in NA19240 and 1.4 Mb in NA12878 (Table 1). The difference in size is partially due to V(DJ) rearrangements and large deletions present in NA12878 relative to NA19240 (Figure 3).

We next validated the accuracy of these assemblies using several orthogonal datasets: Sanger- and SMRT-sequenced fosmid clones, and paired-end Illumina data (Table 1). The Sanger-sequenced fosmids (2) (*n* = 6, NA19240; *n* = 2, NA12878) spanned 210.4 Kb of the NA19240 IGenotyper assembly and 70.2 Kb of the NA12878 assembly (Supplementary Table S9). The percent identity relative to the Sanger-sequenced fosmids was 99.989% for NA19240 and 100% NA12878. We also compared IGenotyper assemblies to additional fosmid assemblies in these samples (Rodriguez et al., unpublished data) sequenced using SMRT sequencing (*n* = 85, NA19240; *n* = 73, NA12878). These collectively spanned 1.5 Mb (82%; NA19240) and 1.2 Mb (82%; NA12878) of the IGenotyper assemblies, aligning with 99.996 and 99.995% sequence identity, respectively (Table 1). The numbers of putative indel errors were 276 (NA19240) and 188 (NA12878) (Supplementary Table S5). Of these indels, 254/276 and 180/188 were 1–2 bp indels, 71.26% (181/254) and 63.33% (114/180) of which were within homopolymers (Supplementary Table S6). Finally, we additionally assessed assembly accuracy using publicly available 30× PCR-free paired-end Illumina TruSeq data from NA19240 and NA12878 (34, 53). We observed a total of 25 discordant bases and 143 indels in the NA19240 assembly (accuracy = 99.989%), and 45 discordant bases and 154 indels in the NA12878 assembly (accuracy = 99.991%; Table 1).

### Assessing Local Phasing Accuracy and Extending Haplotype-Specific Assemblies With Long-Range Phasing Information

To assess local phasing accuracy, we also profiled the IGH locus in the parents of NA19240 and NA12878 (Supplementary Table S4). IGenotyper uses read-backed phasing to delineate reads within haplotype blocks prior to assembly. We tested the accuracy of local phasing (variant phasing within each haplotype block) by comparing read-backed and trio-based phased genotypes. No phase-switch errors were observed in the heterozygous haplotype blocks (*n* = 20 blocks, NA19240; *n* = 24, NA12878). Within homozygous blocks (excluding known SV sites), 27/57,313 (0.05%; NA19240) and 23/139,029 (0.02%; NA12878) bases did not follow a Mendelian inheritance pattern (Supplementary Table S10).

In both NA19240 and NA12878, we observe low localized read coverage in various regions of the locus, representing known technical limitations of probe-based DNA capture (54). Because of this, as well as regions of homozygosity/hemizygosity, IGenotyper is limited in its ability to generate phased haplotype assemblies across the entirety of the locus. However, we reasoned that with long-range phase information (e.g., trio genotypes) contigs from an IGenotyper assembly can be assigned to parental haplotypes. To assess this, heterozygous SNVs in NA19240 and NA12878 were phased using both sequencing reads and parental SNVs, resulting in completely phased haplotypes. To determine potential impacts on accuracy using either the local or long-range phasing approach, we compared each assembly type in the probands. Only 12 (NA19240) and 7 (NA12878) base differences were found between the locally phased and long-range phased assemblies. Taken together, these data suggest that individual contig assemblies generated by IGenotyper have high phasing accuracy.

We anticipate that alternative forms of long-range phasing data will be available in the future. One example would be IGHV, IGHD, and IGHJ haplotype information inferred from AIRR-seq data (17, 18). We assessed whether AIRR-seq based haplotype inference could be theoretically applied, by identifying the number haplotype blocks harboring heterozygous IGHV gene segments. In NA19240 and NA12878, 10/20 and 6/24 of the assembled heterozygous contig blocks harbored at least one heterozygous IGHV gene. In total, this equated to 53.5% (442,057 bps) in NA19240 and 80.72% (342,942 bps) in NA12878 of heterozygous bases that could theoretically be linked using this type of coarse long-range phase information, highlighting the potential strength of pairing the two approaches in larger numbers of samples.

### IGenotyper Produces Accurate Gene Annotation, SNV, Indel, and SV Variant Call Sets From Diploid Assemblies

Previous studies have demonstrated that assembling diploid genomes in a haplotype-specific manner increases the accuracy of variant detection (34, 35, 39, 50, 55–57) and facilitates greater resolution on the full spectrum of variant classes (58). In addition to IGH locus assembly, IGenotyper detects SNVs, short indels, and SVs, including SNV calls within previously characterized complex SV/insertion regions. IGenotyper also provides direct genotypes for five previously described biallelic SVs (see Supplementary Table S11). This excludes the structurally complex *IGHV3-30* gene region, known to harbor multiple complex haplotypes; however, IGenotyper assemblies can be used for manual curation of this region (see below).

Using the fully phased diploid assemblies from each proband (Figure 3), we assessed the validity of annotations/variant calls. We compared proband gene annotations and variant call sets to fosmid and parental assembly data (Table 2). In each sample, we noted the presence of a V(D)J recombination event on one chromosome, which resulted in the artificial loss of alleles (Figure 3A). However, because these events were detectable, they did not preclude our ability to make accurate annotations and variant calls.

**TABLE 2:** Count of different variants identified by IGenotyper.

**Sample**	**Variant type**	**Count**	**Validation rate**
NA19240	SNV	2,912	98.5%(2,869/2,912)
	Indel*	49	100%(49/49)
	SV	11	100%(11/11)
	Reference-embedded SVs	5	100%(5/5)
NA12878	SNV	2,329	99.1%(2,308/2,329)
	Indel*	36	97.2%(35/36)
	SV	3	100%(3/3)
	Reference-embedded SVs	2	100%(2/2)

* Indels greater than 2 bp.

In NA19240, IGenotyper identified 79 unique non-redundant alleles across 57 IGHV genes (Figure 3B and Supplementary Tables S12 and 13); 12 of these alleles were not found in IMGT, representing novel alleles. All 79 alleles were validated by parental and/or fosmid assembly data (Supplementary Table S12). In NA12878, 56 non-redundant alleles were called at 44 IGHV genes (Figure 3B and Supplementary Tables S14, 15), three of which were novel; all 56 alleles were validated (Supplementary Table S14).

Across IGHV we identified 2,912 SNVs, 49 indels (2–49 bps), and 11 SVs (>50 bps) in NA19240. Collectively, IGenotyper-based genotypes for the parents of NA19240 and/or genotypes from the fosmids supported 2,869/2,912 SNVs, 31/36 indels, and 11/11 SVs in NA19240. In NA12878, we identified 2,329 SNVs, 36 indels (2–49 bps), and 3 SVs (>50 bps), 2,308 (SNVs), 20 (indels), and 3 (SVs) of which were supported by orthogonal data. Included in the SVs called from both probands were events within previously identified SV regions (Figure 1A and Supplementary Table S11). All of these regions are polymorphic at the population level (2, 20, 28), and several involve complex duplications and repeat structures (Figure 3A). Strong concordance was observed in these regions between proband IGenotyper assemblies, fosmid clones, and parental CCS reads/assemblies (Supplementary Figures S8–S13 and Table S16).

Additionally, we discovered novel SVs in both NA12878 and NA19240 within the region spanning the genes *IGHV4-28* to *IGHV4-34* (Figure 3A). This site is a known hotspot of structural polymorphisms, in which six SV haplotypes have been fully or partially resolved (2). The longest haplotype characterized to date (2) contains four ∼25 Kb segmental duplication blocks. The novel SV haplotype in NA12878 contains a single ∼25 Kb segmental duplication block, and lacks 6 of the functional/ORF IGHV genes in this region. The novel SV haplotype in NA19240 contains 3/4 segmental duplication blocks, only lacking the genes *IGHV4-30-4* and *IGHV3-30-5*. Both of these novel SVs are supported by fosmid clones and parental data (Supplementary Figure S11).

### Identifying False-Negative and -Positive IGH Variants in Public Datasets

**FIGURE 4:**
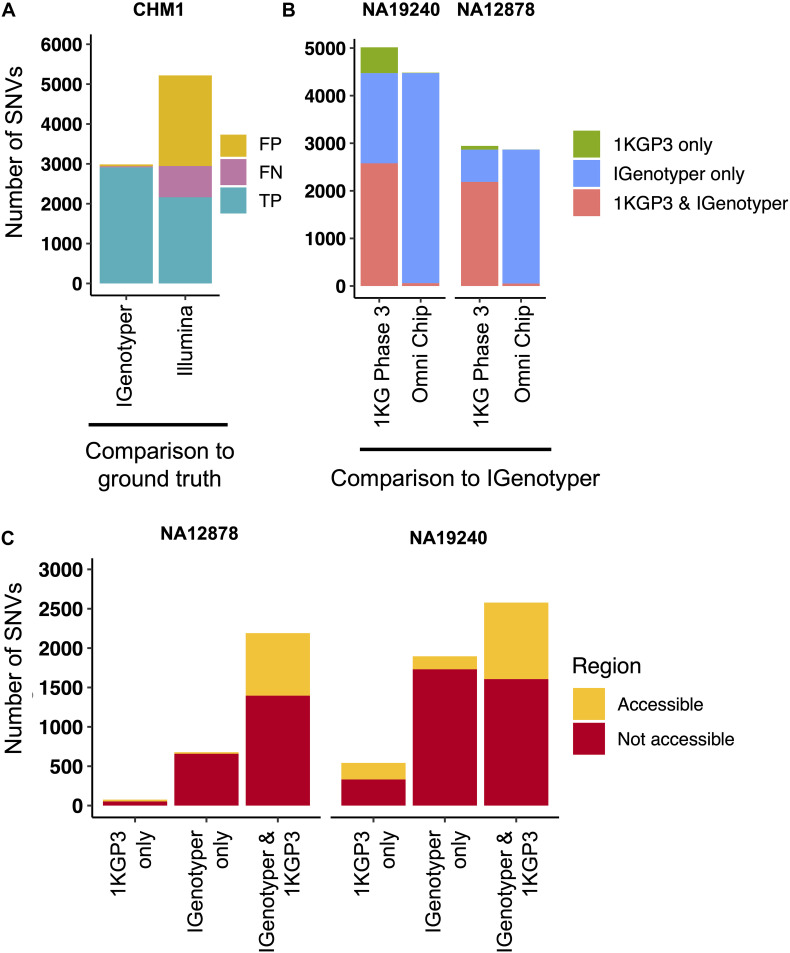
Comparison of SNVs identified by IGenotyper to SNVs called using short-read and microarray data. **(A)** SNVs detected by IGenotyper and Illumina/GATK in CHM1 were compared to a CHM1 ground truth SNV dataset; numbers of false-negative, false-positive, and true-positive SNVs in each callset are shown. **(B)** SNVs in the 1KGP Phase 3 (1KGP3) datasets were compared to SNVs detected by IGenotyper in NA19240 and NA12878. The total number of SNVs in each bar sums to the number of overlapping SNVs and the number of SNVs unique to each dataset. **(C)** SNVs found by IGenotyper and the 1KGP dataset, found only by IGenotyper and found only in the 1KGP dataset were partitioned into regions identified as accessible by the 1KGP accessible genome browser track.

Pitfalls of using short-read data for IGH variant detection and gene annotation have been discussed previously (3, 59). We assessed potential advantages of our approach compared to other high-throughput alternatives. In the CHM1 dataset, we defined a ground truth IGH SNV dataset by directly aligning the IGH locus haplotype from GRCh38 (2) to that of GRCh37 (1). We identified 2,940 SNVs between the two haplotypes in regions of overlap (i.e., non-SV regions). We next aligned Illumina paired-end sequencing data generated from CHM1 (60) and our CHM1 IGenotyper assemblies to GRCh37. We detected 4,433 IGH SNVs in the Illumina dataset, and 2,958 SNVs in the IGenotyper assembly. The Illumina call set included only 73.2% (2,153) of the ground truth SNVs, as well as an additional 2,274 false-positive SNVs (Figure 4A). In contrast, the IGenotyper call set included 99.0% (2,912) of the ground truth SNVs, and only 46 (1.6%) false-positive SNVs were called (Figure 4A).

We next compared IGenotyper genotypes for NA19240 and NA12878 to 1KGP short-read and microarray data (Figure 4B). IGenotyper SNVs were lifted over to GRCh37 (*n* = 4,474, NA19240; *n* = 2,868, NA12878), excluding SNVs within SV regions not present in GRCh37 (*n* = 703, NA19240; *n* = 737, NA12878) Supplementary Table S17). In total, only 57.6% (2,578/4,474) and 76.4% (2,190/2,868) of the IGenotyper SNVs were present in the 1KGP call set for NA19240 and NA12878 (Figure 4B and Supplementary Table S18). Critically, because insertion SVs are not present in GRCh37, the additional SV-associated SNVs were also missed. Thus, in total, 50.2% (2,599/5,177) and 39.3% (1,415/3,605) of IGenotyper SNVs were absent from 1KGP (Figure 4B), including SNVs within 18 and 6 IGHV genes; 1,350/4,474 (NA19240) and 526/2,868 (NA12878) IGenotyper SNVs were not found in any 1KGP sample. The 1KGP call set also included an additional 542 (17.4%) and 76 (3.4%) SNVs (putative false-positives) for NA19240 and NA12878, respectively, including putative false-positive SNVs in 6 and 3 IGHV genes (Figure 4B). In contrast to SNVs found only in the 1KGP datasets, we found that in both probands >90% of SNVs detected only by IGenotyper were within short read-inaccessible regions (NA19240, 91.3%, 1,731/1,896; NA12878, 97.1%, 658/678; Figure 4C and Supplementary Table S19), suggesting that IGenotyper offers improvements in regions that are inherently problematic for short reads. We additionally assessed HWE at the interrogated SNVs, as deviation from HWE is often used to assess SNV quality. In both probands, we found that a greater proportion of SNVs unique to the 1KGP callset deviated from HWE than those called by IGenotyper (Supplementary Figure S14).

Finally, we compared larger variants, indels and SVs identified by IGenotyper to those detected by the Human Genome Structural Variation Consortium (HGSV) in NA19240 (34). First, we assessed support for six large known identified SVs in NA19240 (Figure 3A and Supplementary Tables S11 and S21). BioNano optical mapping data detected events in five out of the six SV regions. The complex SV spanning *IGHV1-8*/*3-9*/*IGHV3-64D*/*5-10-1* was not detected, likely because this event involves a swap of sequences of similar size (∼38 Kb) (2), making it difficult to identify using BioNano. A critical difference to BioNano is that IGenotyper provides nucleotide level resolution allowing for fuller characterization of SV sequence content, including SNVs within these regions (as noted above). In addition to these large SVs, IGenotyper also identified 39 indels (3–49 bps) and an additional 11 SVs (>49 bps; 57–428 bps) in NA19240. Of these, 20 indels and 9 SVs were present in the HGSV integration call set.

### Effects of False-Positive and -Negative Variants on Imputation Accuracy

**FIGURE 5:**
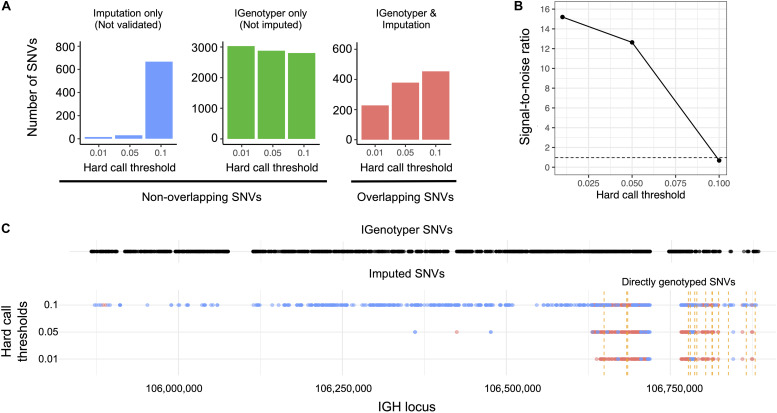
Comparison of IGenotyper variant calls to microarray-/imputation-based genotyping. **(A)** SNVs detected by IGenotyper were compared to array-based and imputed SNVs in a sample from a recent RHD GWAS. Imputed SNVs were filtered based on different hard call thresholds (0.01, 0.05, and 0.1). Each set of filtered SNVs was compared to IGenotyper SNVs. The number of imputed SNVs not identified by IGenotyper (blue), SNVs only detected by IGenotyper (green), and overlapping SNVs (red) are shown above. **(B)** The signal-to-noise ratio was calculated using the overlapping SNVs between IGenotyper and imputation, and the SNVs identified only by imputation (not called by IGenotyper). Below the signal-to-noise ratio of 1 (dotted line), there is more noise than signal. **(C)** Positions of SNVs detected by IGenotyper and at different hard call thresholds. Each point represents a SNV. Blue SNVs are SNVs only identified by imputation and not detected by IGenotyper, and red SNVs are SNVs found both by imputation and IGenotyper.

We explored the potential advantage of our genotyping approach compared to array genotyping and imputation. We applied our long-read capture method to a sample selected from a recent rheumatic heart disease (RHD) GWAS (24), which identified IGH as the primary risk locus. Direct genotyping in this sample was carried out previously using the HumanCore-24 BeadChip (*n* = 14 SNVs) and targeted Sanger sequencing (*n* = 8 SNVs); genotypes at additional SNVs were imputed with IMPUTE2 (61), using a combination of 1KGP and population-specific sequencing data as a reference set. We compared IGenotyper SNVs from this sample to directly genotyped variants and imputed variants selected at three hard call thresholds (0.01, 0.05, and 0.1; Figure 5). The majority of directly genotyped SNVs (array, 13/14; Sanger, 8/8) were validated by IGenotyper. However, the validation rate varied considerably for the imputed SNVs, depending on the hard call threshold used (Figure 5A; 93.8%, 0.01; 92.7%, 0.05; 40.5%, 0.1), with the signal to noise ratio decreasing significantly from the 0.01 to the 0.1 threshold (Figure 5B). In all cases, IGenotyper called a substantial number of additional SNVs (Figure 5A); the majority of these were located in the proximal region of the locus, which was poorly represented by both directly genotyped and imputed SNVs (Figure 5C).

### Sample Multiplexing Leads to Reproducible Assemblies and Variant Calls

**FIGURE 6:**
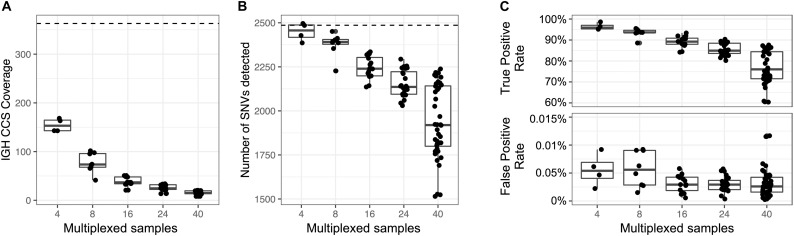
Assessment of sample multiplexing on assembly coverage and variant calling. Eight replicates of NA12878 were multiplexed and sequenced on a single Sequel SMRT cell. The eight replicates were combined or down sampled to simulate a sequencing run with 2, 4, 16, 24, and 40 samples. The simulated sample from the 2-plex run with the highest coverage was treated as the ground truth. **(A)** CCS coverage of sequencing runs with different numbers of multiplexed samples. The dotted line represents the coverage of a sample from a 2-plex sequencing run. **(B)** Number of SNVs found across samples per each multiplexed sequencing run. The dotted line represents the number of SNVs detected in a sample from a 2-plex sequencing run. **(C)** True and false positive rate of SNVs of each sample in each multiplexed sequencing run. The SNVs from each sample were compared to SNVs from a sample sequenced in a 2-plex run.

An advantage to the capture-based approach employed here is the ability to multiplex samples. To demonstrate this, eight technical replicates of NA12878 were captured, barcoded, pooled, and sequenced on a single Sequel SMRT cell 1M (Supplementary Table S22), yielding a mean CCS coverage ranging from 41.3 to 101.6x for each library (Figure 6A). We then simulated different plexes (2-, 4-, 16-, 24-, 40-plex) by either combining or partitioning data from this 8 plex, allowing us to assess the impacts of read depth on IGH locus coverage, assembly accuracy, and variant calling. The mean CCS coverage per plex ranged from 308.7X (2-plex) to 15.5X (40-plex) (Figure 6A). To compare IGenotyper metrics across plexes, we chose the 2-plex sample with the highest CCS coverage to use as the ground-truth dataset; all other assemblies and variant call sets were compared to this sample. The lower coverage 2-plex assembly covered 99.75% of the ground truth assembly with a sequence identity concordance of 99.99%. For the remaining comparisons, mean assembly coverage and sequence concordance estimates ranged from 99.27% (4-plex) to 86.95% (40-plex), and 99.99% (4-plex) to 99.99% (40-plex). The mean number of observed SNVs ranged from 2,471 (2-plex) to 1,936 (40-plex) (Figure 6B). When comparing these to ground truth SNVs, we observed high recall rates (>80%), even among the 40-plex assemblies (Figure 6C); recall was >90% for all but one of the 4- and 8-plex assemblies (Figure 6C). Importantly, although the recall rate of true-positive SNPs decreased as expected in higher plexes, we observed very little variation in the false-positive rate (Figure 6C).

## Discussion

For decades, comprehensive genetic analysis of the human IGH locus has been intractable (3, 23, 59). As a result, our understanding of the extent of IGH germline diversity in human populations, and how this diversity contributes to B cell mediated immunity remains incomplete (3, 23). Here, we have leveraged existing genomic haplotype data to design a novel IGH assembly and genotyping framework that combines targeted long-read sequencing with a novel bioinformatics toolkit (IGenotyper). This end-to-end pipeline can reconstruct completely phased assemblies via the integration of long-range phase information. Utilizing high-quality CCS reads and derived assemblies facilitates characterization of IGH gene segments and all forms of coding and non-coding variants, including the discovery of novel variants and IG alleles.

We validated our pipeline on eight ethnically diverse human samples with orthogonal data that highlighted multiple strengths of our approach. First, we chose individuals with available BAC and fosmid clone-based assembly datasets for direct comparisons to capture/IGenotyper assemblies, as well as Illumina short-read data for assessing assembly error correction metrics. These comparisons revealed high concordance between assemblies (>99%) in both haploid and diploid samples. Second, we directly validated variants and alleles using trio data. Finally, comparisons to additional variant call sets (1KGP and HGSV) allowed us to assess concordance in variant detection (including indels and SVs), and demonstrate advantages over alternative high-throughput methods. Specifically, compared to microarray-based and short-read sequencing methods, IGenotyper variant call sets were more comprehensive, exhibited greater locus coverage, and were more accurate.

Much recent effort has focused on identifying IG genes/alleles absent from existing databases (2, 5–8, 11, 19, 62–65), revealing many undiscovered alleles in the human population. In our analysis, we identified 15 novel alleles from only two samples. Consistent with previous suggestions of undersampling in non-Caucasian populations (7), the majority (*n* = 12) were in the Yoruban individual, for which 6 additional novel alleles had been reported in an earlier study (1, 2). Notably, the novel alleles described in NA19240 represent the largest contribution to the IMGT database from a single individual. Related to this point, we also observed high numbers of false-positive/negative IGHV SNVs in 1KGP datasets, reinforcing that efforts to identify IG alleles from 1KGP data should be done with extreme caution (59, 66, 67). An added advantage of our approach is the ability to capture variation outside of IG coding segments and more fully characterize SVs. Although several studies have begun to demonstrate the extent of SV haplotype variation (2, 17, 18, 65), information on polymorphisms within these SVs, and within IGH regulatory and intergenic space remains sparse (23). It is worth noting that, in the few samples analyzed here, the majority of variants were detected in non-coding regions, including SNVs within RS, leader, intronic, and intergenic sequences. We also showed that IGenotyper resolved novel SVs within the complex *IGHV3-30* gene region in both 1KGP diploid samples. Together, these examples are testament to the fact that our approach represents a powerful tool for characterizing novel IGH variation.

Despite evidence that IG polymorphism impacts inter-individual variation in the antibody response (13, 20, 21, 27), the role of germline variation in antibody function and disease has not been thoroughly investigated. The population-scale IGH screening that will be enabled by this approach will be critical for conducting eQTL studies and integrating additional functional genomic data types to better resolve mechanisms underlying IG locus regulation, which have only so far been applied effectively in model organisms (68–71). Delineating these connections between IGH polymorphism and Ab regulation and function will be critical for understanding genetic contributions to Ab mediated clinical phenotypes (23).

To date, few diseases have been robustly associated to IGH (24, 25, 72, 73). We previously suggested this was due to sparse locus coverage of genotyping arrays and an inability of array SNPs to tag functional IGH variants (2, 3). We have provided further support for this idea here. First, the identification of both putative false negative and positive SNVs in 1KGP samples highlights potential issues with imputation-based approaches using 1KGP samples as a reference set. Second, our direct analysis of capture/IGenotyper data in a sample from a recently conducted GWAS (24) also demonstrated that IGenotyper resulted in a larger set of genotypes, with improved locus coverage compared to imputation. Together, these analyses highlight the potential for our framework to supplement GWASs for both discovery and fine mapping efforts, and through building more robust imputation panels. A strength of our approach is that the user can determine the sequencing depth and locus coverage, depending on whether the intent is to conduct full-locus assemblies or genotyping screens; although the number of detected variants decreases with increased multiplexing, false-positive rates remain low. The recently released, higher throughput Sequel II platform, in combination with read length improvements, will allow for expanded processing of larger cohorts at lower cost.

A current technical limitation of our framework is the decreased efficiency of probes in particular regions of IGH. However, we showed that these regions represent a small fraction of IGH, with overall negligible impacts on locus coverage and assembly quality. Future iterations of target-enrichment protocols will improve efficiency through methodological or reagent modifications. A key strength of IGenotyper is its flexibility to accommodate other data types; e.g., users interested in complete haplotype characterization can already provide long-range phase information to inform a complete diploid assembly. We envision other forms of data will be leveraged in future applications. A second limitation is the potential to miss unknown sequences not specifically targeted by capture probes. We expect this issue to be mitigated in the future as more IG haplotypes are sequenced using the method described here, as well as through the application of large-insert clone assembly and WGS approaches; enumeration of these data will allow for refinement on protocol design and IGenotyper functionality. Ultimately, we promote an advance toward a more extensive collection of IGH haplotype reference datasets and variants as a means to leverage more sophisticated strategies for variant calling; for example, population reference graph approaches, which have been shown to be effective in other hyperpolymorphic immune loci (74). Looking forward, we expect our approach will lead to more comprehensive datasets that will augment and extend existing IG germline databases, such as IMGT (4) and VDJbase (75), and facilitate more effective modes of sharing IG polymorphism and haplotype data.

To the best of our knowledge, this is the first combined molecular protocol and analytical pipeline that can provide comprehensive genotype and annotation information across the IGH locus, with the added ability to be applied to a large number of samples in a high-throughput manner. Given the importance of antibody repertoire profiling in health and disease, characterizing germline variation in the IG regions will continue to become increasingly important. Our strategy moves toward the complete ascertainment of IG germline variation, a prerequisite for understanding the genetic basis of Ab-mediated processes in human disease.

## Data Availability Statement

All raw data and fosmid assemblies used to validate our approach can be found under BioProject PRJNA555323.

## Ethics Statement

Ethical approval was granted previously for the usage of the RHD GWAS study sample by the Hospital Ethics Committee at the Hôpital de Gaston-Bourret and the Comité d’Evaluation Ethique de l’Inserm as well as the Oxford University Tropical Research Ethics Committee. Previous consent was granted for the hydatidiform mole sample procured from the laboratory of Dr. Urvashi Surti. The sample was obtained from the participant with written informed consent to be used for research purposes.

## Author Contributions

OLR, WSG, AJS, MLS, AB, and CTW conceived and planned the study. OLR and TP performed the computational analyses. OLR and CTW wrote the manuscript with contributions from TP, EEE, AJS, WSG, MLS, and AB. OLR wrote the code. EEE, KA, and TP provided the additional samples. WSG, ME, and MLS prepared the sequencing libraries. GD, JP, MLS, and RS performed the sequencing. CTW, AB, and MLS supervised the experiments, analysis, and data interpretation. All authors read and approved the final manuscript.

## Conflict of Interest

EE is on the Scientific Advisory Board (SAB) of DNAnexus, Inc.

The remaining authors declare that the research was conducted in the absence of any commercial or financial relationships that could be construed as a potential conflict of interest.

The reviewer GY declared a past co-authorship with one of the authors, CW, to the handling editor.

## Footnotes

1 https://igenotyper.github.io/

2 https://github.com/PacificBiosciences/ccs

3 http://igenotyper.github.io/
