# Supplementary material for: A Novel Framework for Characterizing Genomic Haplotype Diversity in the Human Immunoglobulin Heavy Chain Locus
Source: Front Immunol. 2020 Sep 23;11:2136. doi: 10.3389/fimmu.2020.02136 (PMC7539625; doi:10.3389/fimmu.2020.02136)
Supplement: Supplementary file 1 [file Table_1.pdf]

Supplementary Text

Methods

Fig. S1. HTML summary report from IGenotyper.

Fig. S2. Read length and average base call accuracy across CCS reads in CHM1.

Fig. S3. Three large discrepancies in CHM1 and NA19240 IGenotyper assemblies are expansions of 59mer tandem repeat motif.

Fig. S4. Parent-child trios used in study.

Fig. S5. V(D)J recombination in NA19240

Fig. S6. V(D)J recombination in NA12878

Fig. S7. Assembling all reads in regions of homozygosity

Fig. S8. Validation of insertion with IGHV7-4-1 gene in NA19240

Fig. S9. Validation of complex structural variant with IGHV1-8 and IGHV3-9 genes in NA19240

Fig. S10. Validation of duplication with IGHV3-23D gene in NA19240

Fig. S11. Validation of previously detected duplications harboring IGHV4-28, IGHV3-30, IGHV4-30-2, IGHV3-30-3, IGHV4-30-5, IGHV3-30-5, IGHV4-31, IGHV3-33 and IGHV4-34 genes

Fig. S12. Validation of insertion harboring IGHV4-38-23, IGHV3-43D, IGHV3-38-3 and IGHV1-38-4 gene.

Fig. S13. Validation of previously detected insertion harboring IGHV1-69, IGHV2-70D, IGHV1-69-2, IGH1-69D and IGHV2-70 genes.

Fig. S14. Analysis of SNVs in IGH that fail or pass HWE

Table S1. Sequences used to make custom IGH reference.

Table S4. Samples used in this study sequenced with different platforms and panels.

Table S5. Number and total bases of errors from incorrectly inserted sequence and missing sequence (indel errors) in the assembly of CHM1, NA19240 and NA12878.

Table S6. Indel errors in the assemblies separated by size with homopolymer annotation.

Table S7. Coordinates of V(D)J recombination in the two trios whose genomic DNA were derived from LCLs.

Table S8. Number of haplotype blocks and heterozygous blocks

Table S9. Number of fosmids used to validate assemblies.

Table S10. Mendelian inconsistencies rate in homozygous blocks

Table S11. Embedded structural variants in the IG-reference.

Table S12. Alleles for IGHV genes for NA19240 and the inherited alleles in the parents of NA19240 (NA19238 and NA19239)

Table S13. Sequence for novel alleles detected in NA19240

Table S14. Alleles for IGHV genes for NA12878 and the inherited alleles in the parents of NA12878 (NA12892 and NA12891)

Table S15. Sequence for novel alleles detected in NA12878

Table S16. Validation of genotyped structural variants with fosmids and parental assemblies.

Table S17. Number of SNVs lifted over to GRCh37/hg19 in NA19240 and NA12878

Table S18. Number of overlapping SNVs in NA19240 and NA12878 between IGenotyper and the 1KGP phase 3 datasets

Table S19. Number of SNVs within accessible regions defined by 1KGP

Table S20. Number of SNVs passing or failing ($p < 0.001$) Hardy-Weinberg equilibrium

Table S21. Genotypes for NA19240 embedded structural variants in custom IGH reference.

Table S22. Statistics from multiplexing replicates of NA12878

Methods

Description of capture panels

Three different target panels (“A”, ”B” and “C”) were developed and tested. The sequencing probes were developed by providing Roche with a fasta file representing IGH sequence targets. Roche then returned coordinates of targeted regions. Targets for panel A were developed from 12 different fosmid sequences spanning the IGH locus and regions within chromosome 14 of hg38 (Table S1). Targets for panel B were developed from the custom IGH reference we created for IGenotyper (Figure 1a). Targets for panel C were developed from the custom IGH reference and 97 additional regions from hg38. Ninety-six additional regions correspond to Ancestry Informative Markers (AIMs). The last additional region corresponds to the IGHC region. Analysis features for AIMs and the IGHC region are currently not operational in IGenotyper, but will be incorporated into future versions.

In panel B and C, additional probes (boosting) were added to increase the sequencing coverage (Table S3). The amount of boosting ranged from 2 to 5. The mean coverage was consistent in panel B across IGH, we noted a loss in coverage over the IGHJ region in panel A. We speculate this is caused by a lack of adjacent target sequence on the 3’ flank of the IGHJ region in panel A, in contrast to panel B, which also included

sequence targets across the entirety of the IGHC region. For CHM1, we combined data from panels A and B to mitigate inconsistencies in regional coverage between them.

Sequence target files will be made available by request.

Large insertions within the IGenotyper assemblies not found in GRCh38 and fosmids

Haplotype 1 of NA19240 contained a 1,704 bp and 1,456 bp insertion that was not found in the sequenced fosmids. The CHM1 assembly contained a 2,226 bp insertion not found in GRCh38. These were the largest differences that were found between the IGenotyper assemblies and the ground truth sequences. All three large insertions were found within the duplication containing the genes *IGHV1-69, IGHV2-70, IGHV1-69-2, IGHV1-69D* and *IGHV2-70D*. All these insertions contained an expansion of the tandem repeat motif "TTTAAAAAGATAGTTCTCATCGCCTTGAATTGTGGGAGCAGCTCAGATGTGATAGAATA". This tandem repeat has been previously difficult to assemble. The BAC clone CH17-212P11 (AC245369.4) underlying this region in GRCh38 did not resolve this duplication. Under features in the genbank accession, the tandem repeat is labelled as "unresolved duplication". Therefore it is possible that the IGenotyper assembly is correct, and the reference is incorrect.

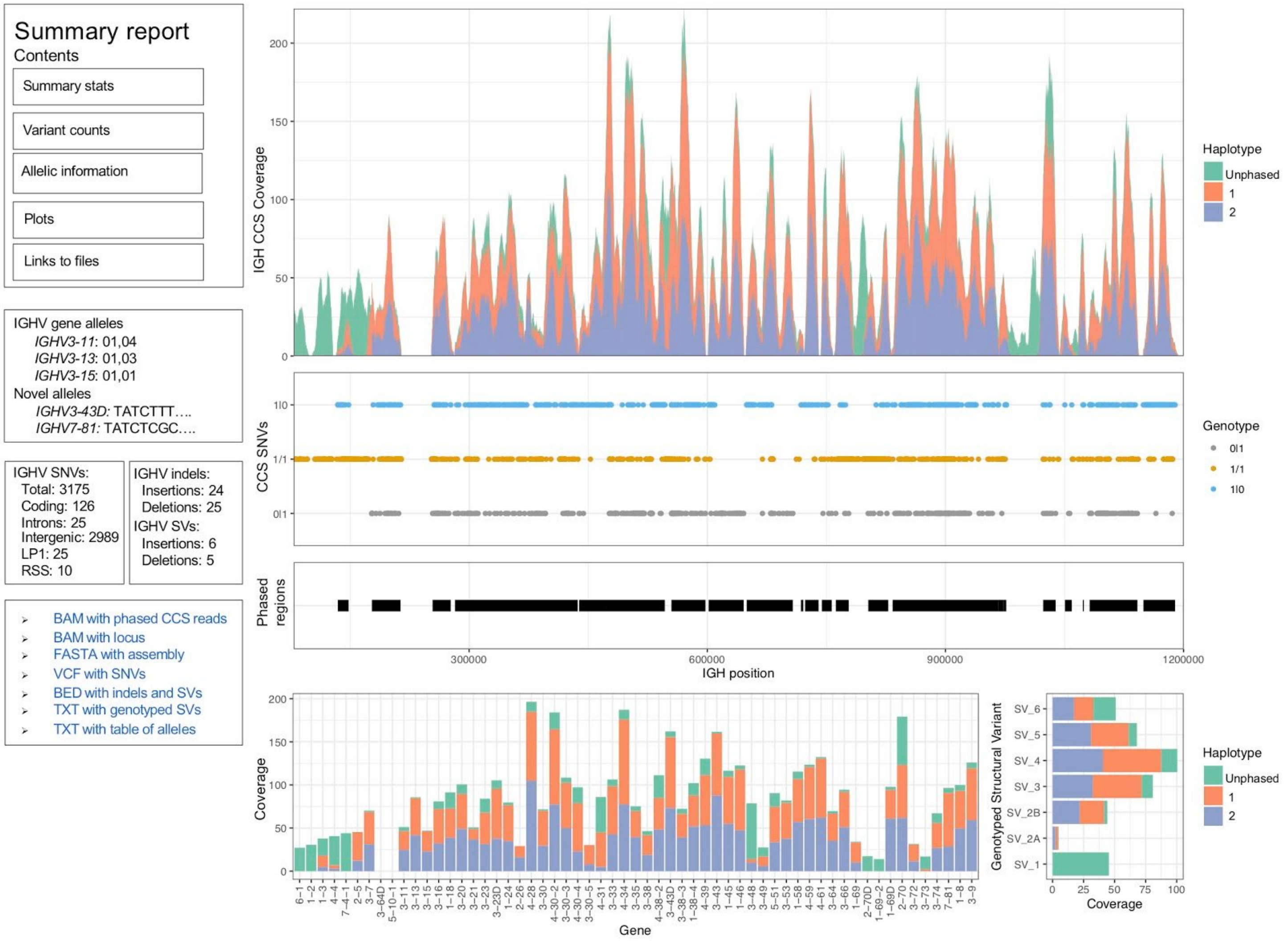

Fig. S1. HTML summary report from IGenotyper.

IGenotyper produces a summary report with tables containing different sequencing and assembly summary statistics, the count of variants (SNVs, indels, and SVs), genotypes of the IGHV,D, and J genes and a link to files with the variants and alleles (VCF, BED and TXT files).

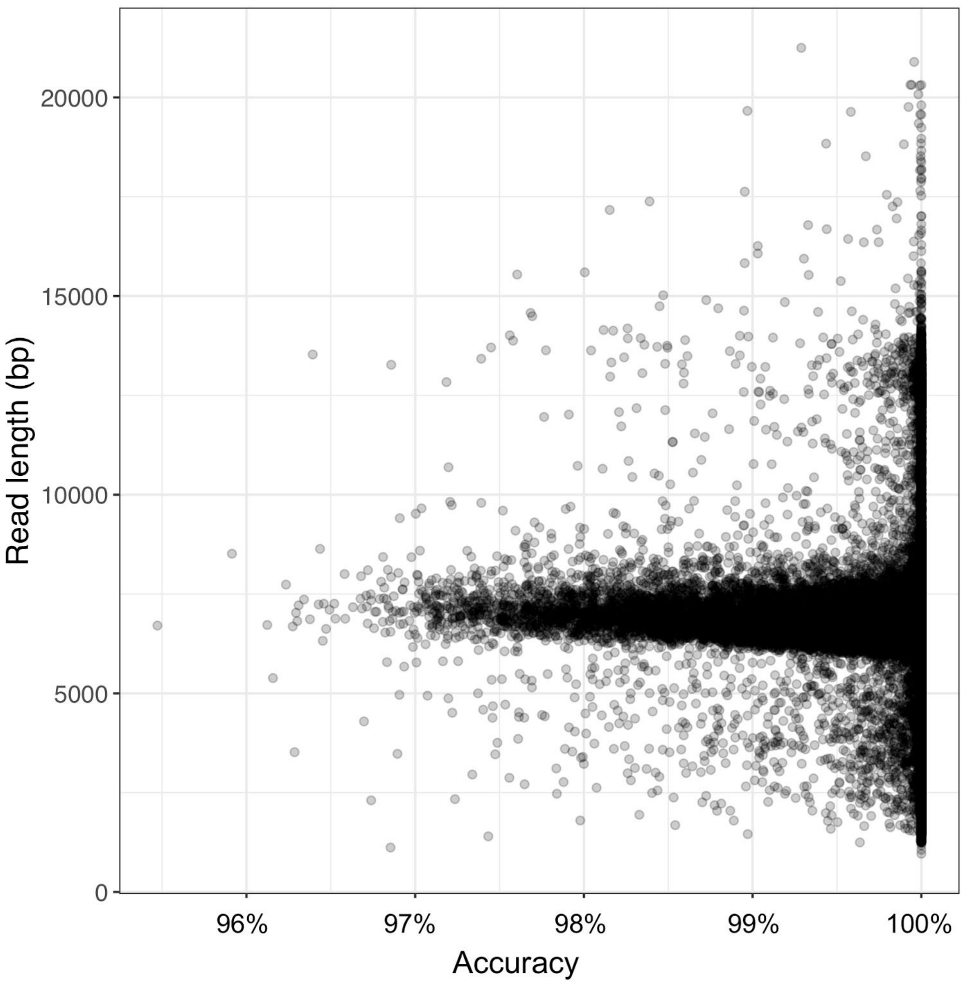

Fig. S2. Read length and average base call accuracy across CCS reads in CHM1.

Every point is a CCS read. Raw SMRT sequences with at least two passes were converted into CCS reads. The phred quality score for every base in the CCS read was averaged. The y-axis has the base call accuracy of the average phred quality score. The x-axis is the length of the CCS read.

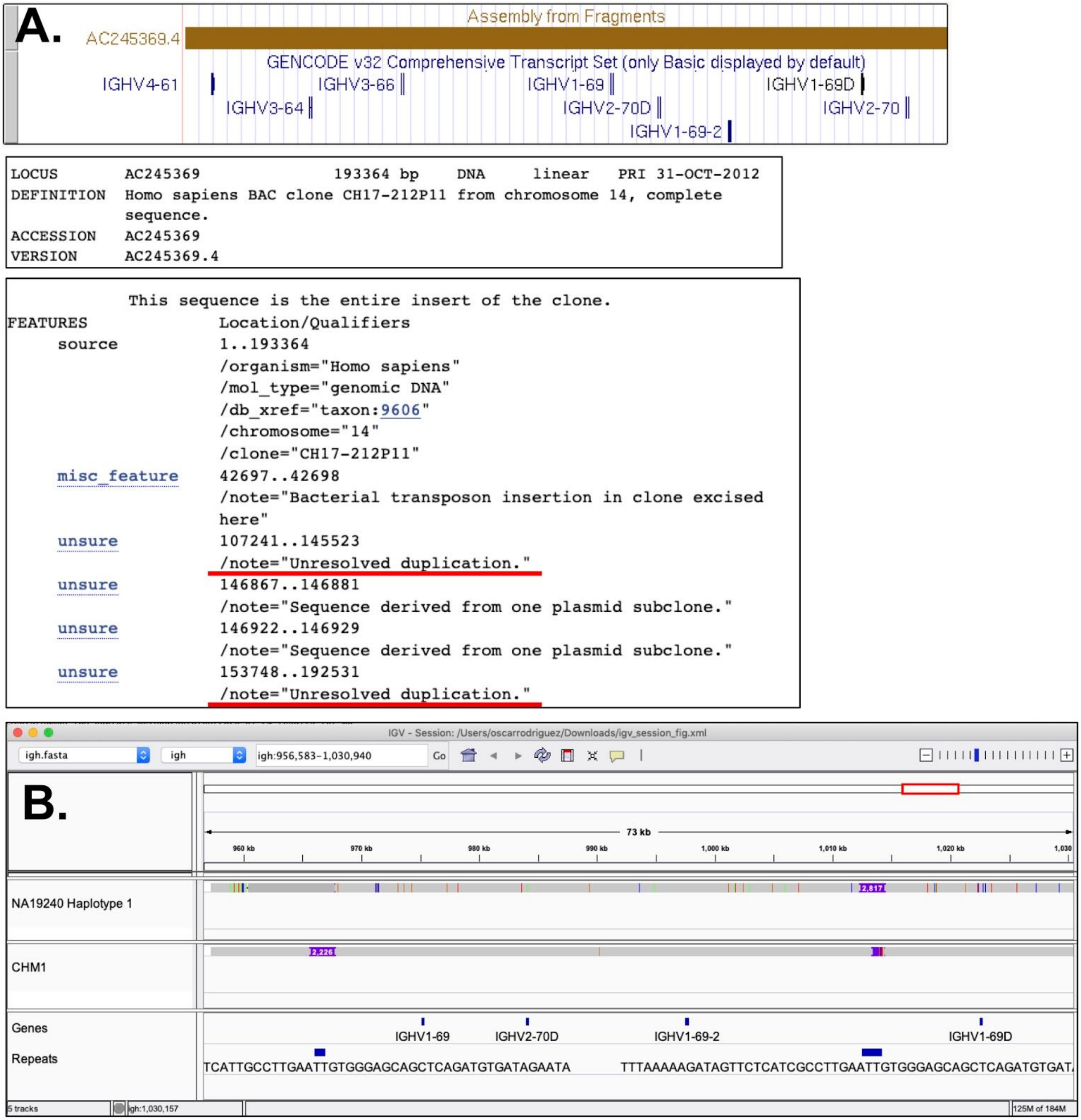

Fig. S3. Three large discrepancies in CHM1 and NA19240 IGenotyper assemblies are expansions of 59mer tandem repeat motif.

(**A**) The BAC clone, AC245369.4, was used to assemble the IGH region containing the genes from *IGHV3-66* to *IGHV2-70*. The BAC clone contained two unresolved duplications (red lines). The unresolved duplications are a tandem repeat with a 56-mer motif

"TTTAAAAAGATAGTTCTCATCGCCTTGAATTGTGGGAGCAGCTCAGATGTGATAGAATA" shown in (**B**).

(**B**) The CHM1 assembly from IGenotyper contained two large insertion sequences of 1,704 bp and 1,456 bp within this tandem repeat. Since the duplication was not resolved in GRCh38, we are uncertain if IGenotyper is correct or not. The NA19240 haplotype 1 also contains an insertion with this tandem repeat in the IGenotyper assembly that does not agree with the NA19240 haplotype 1 fosmids spanning this region. This can be due to misassembly, somatic expansion, or an artifact in the cell line.

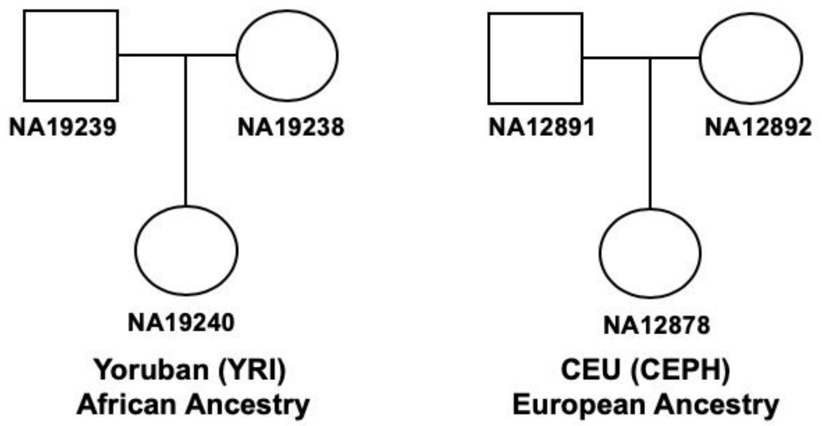

Fig. S4. Parent-child trios used in study.

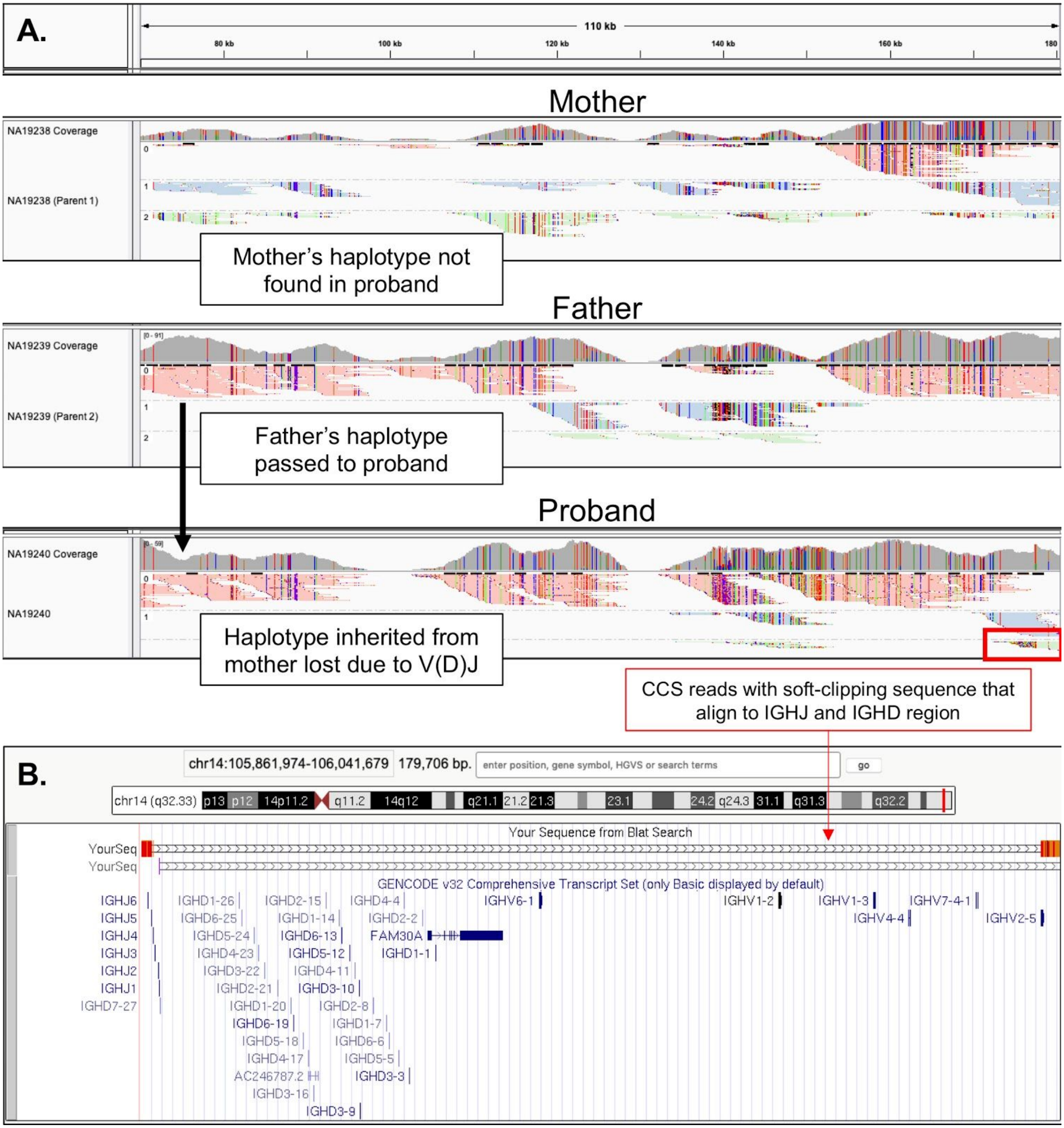

Fig. S5. V(D)J recombination in NA19240

(A) IGV screenshot of CCS reads from NA19240, NA19238 and NA19239 aligned to the proximal region of IGH. NA19240 (proband) lost the beginning part of a single haplotype due to V(D)J recombination. The proband still contains the haplotype inherited from the father (NA19239) but not the mother's inherited

haplotype (NA19238). The mother's inherited haplotype appears after *IGHV2-5* in the proband. CCS reads spanning *IGHV2-5* in the proband are not fully aligned to the reference and contain soft-clipping sequences that align to the D and J genes as shown in the BLAT output in (**B**).

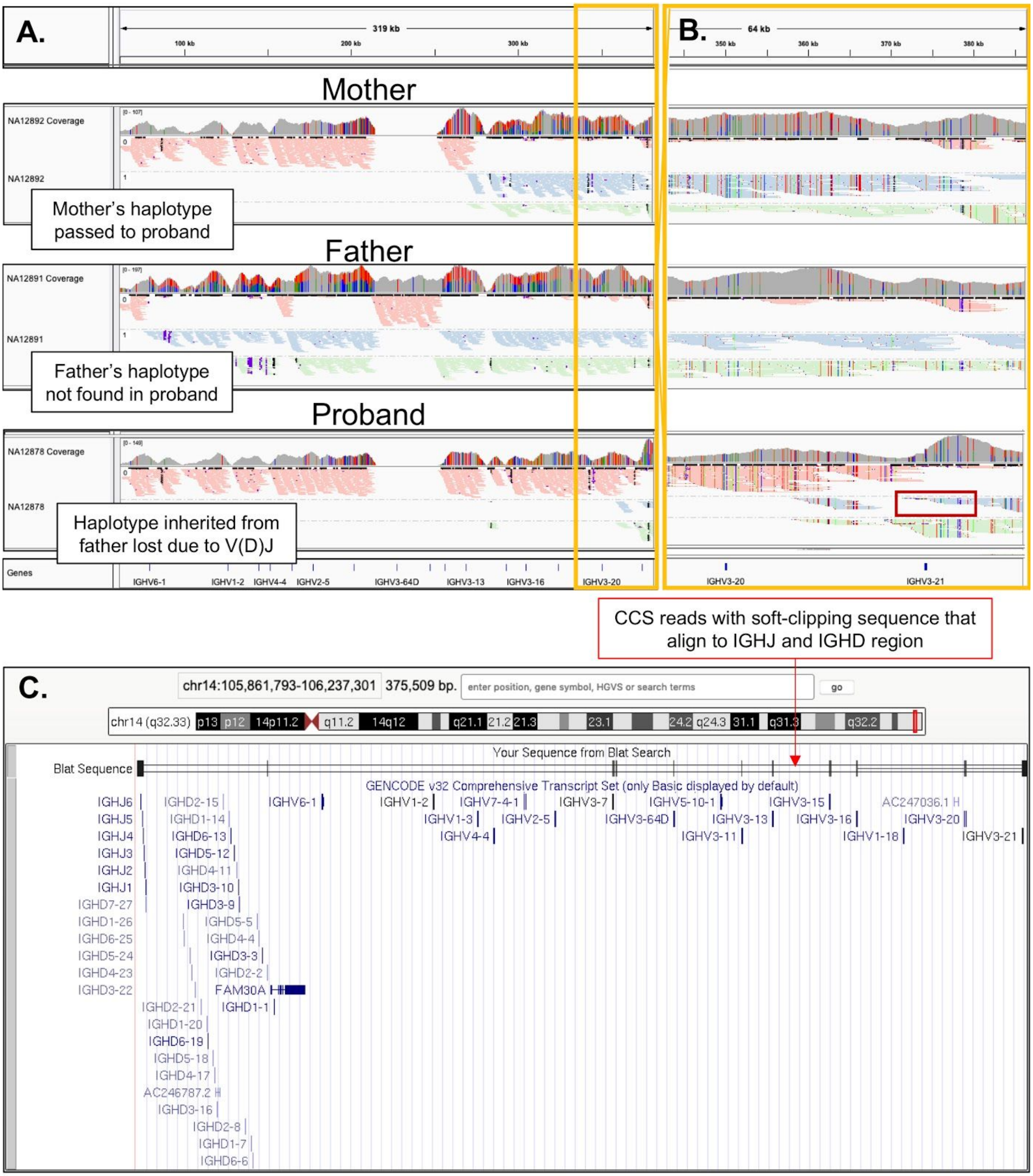

Fig. S6. V(D)J recombination in NA12878

(A,B) IGV screenshot of CCS reads from NA12878, NA12891 and NA12892 aligned to the proximal region of IGH. NA12878 (proband) lost the beginning part of a single haplotype due to V(D)J recombination. The

proband still contains the haplotype inherited from the mother (NA12892) but not the father's inherited haplotype (NA12891). The father's inherited haplotype appears after *IGHV3-21* in the proband. CCS reads spanning *IGHV3-21* in the proband are not fully aligned to the reference and contain soft-clipping sequences that align to the D and J genes as shown in the BLAT output in (**C**).

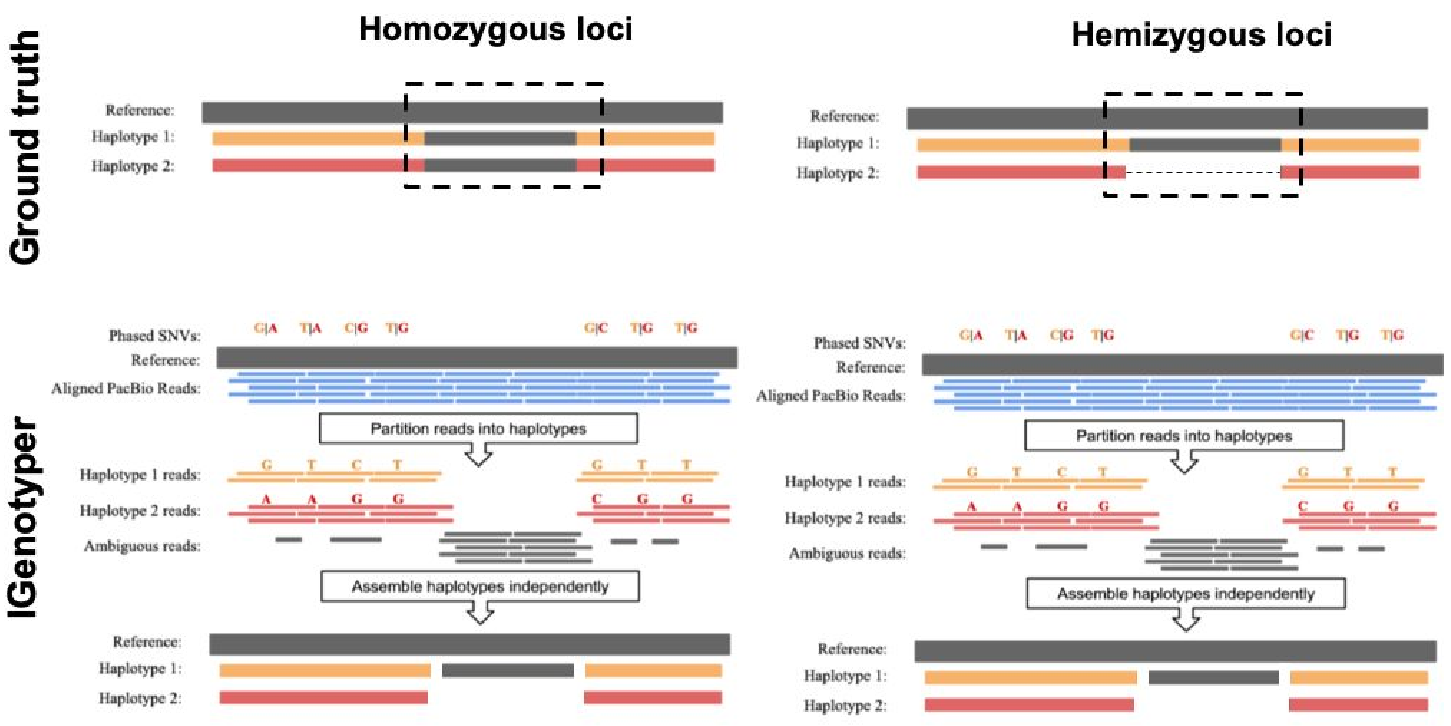

Fig. S7. Assembling all reads in regions of homozygosity

Schematic showing the phased reads and assembly process in a region of homozygosity and hemizygosity. All reads in both cases are assembled together. The assembly process is the same.

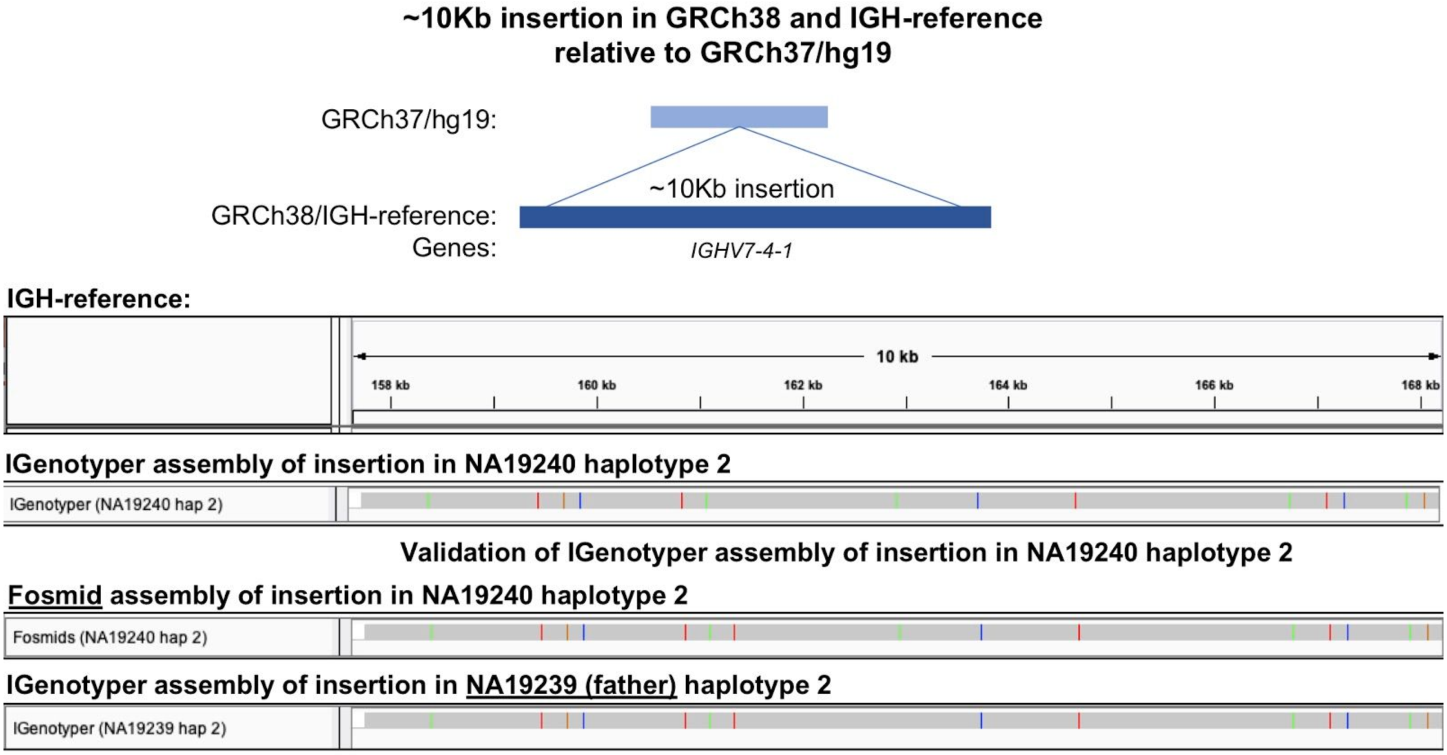

Fig. S8. Validation of insertion with *IGHV7-4-1* gene in NA19240

Top: Schematic showing insertion identified by Watson et al 2013 in CHM1 (GRCh38) relative to GRCh37/hg19. The insertion from CHM1 is present in the IGH-reference.

Bottom: IGenotyper assemblies and fosmids shown in order:

1) NA19240 haplotype 2
2) NA19240 haplotype 2 fosmids
3) NA19239 haplotype 2

NA19240 contained the insertion in a single haplotype. The alternate haplotype was lost due to a V(D)J event. The insertion was validated through fosmids and inheritance with parental data. The assembly of the insertion shows high concordance with sequenced fosmids and the assembled haplotype from NA19239 (father).

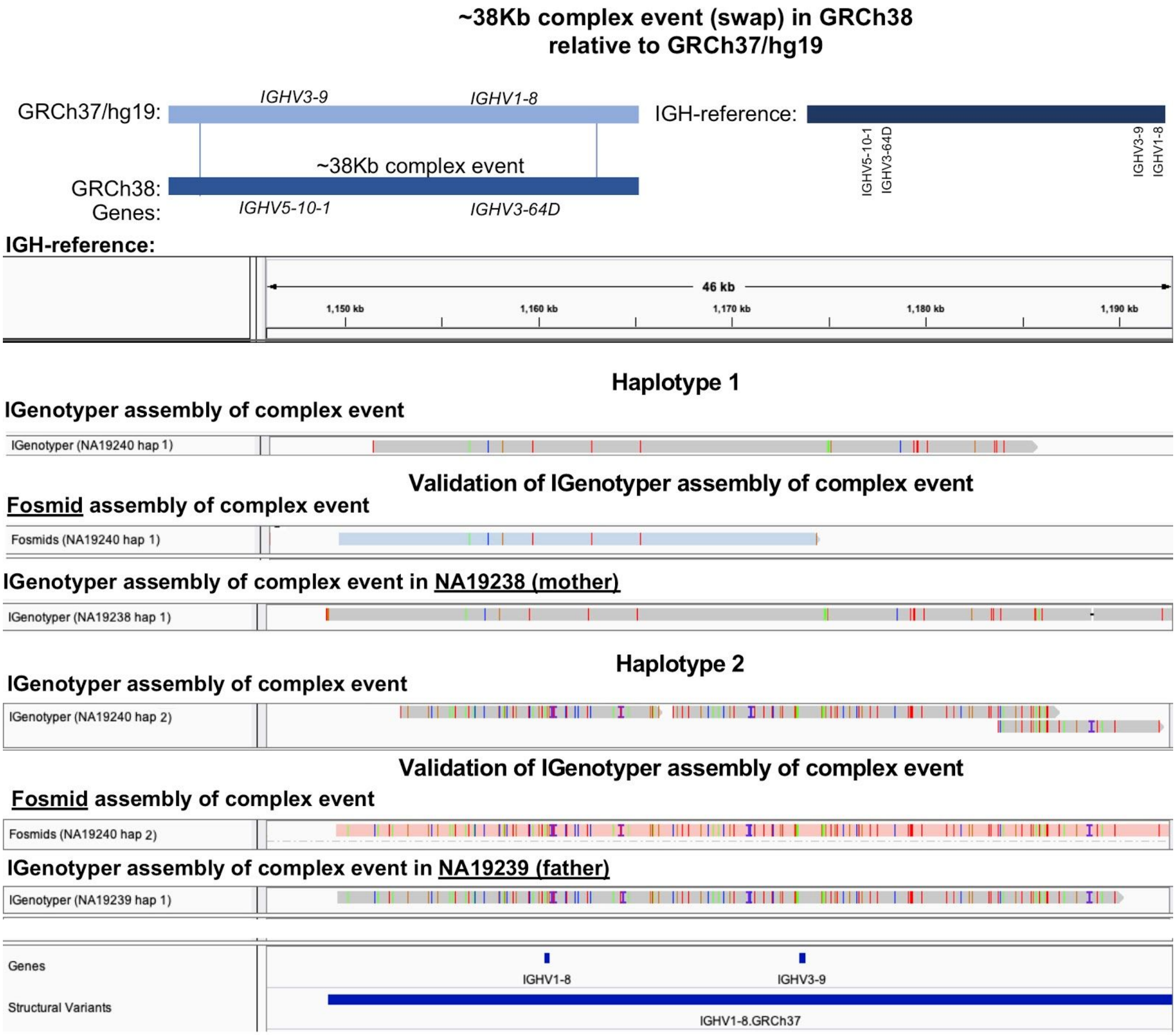

Fig. S9. Validation of complex structural variant with *IGHV1-8* and *IGHV3-9* genes in NA19240

Top: Schematic showing the complex event identified by Watson et al 2013 in CHM1 (GRCh38) relative to GRCh37/hg19. The complex event is not a straightforward insertion or deletion, rather it is a swap of sequence of the same length. The sequence present in GRCh37/hg19 was placed at the end of the IGH-reference (right).

Bottom: IGenotyper assemblies and fosmids shown in order:

1) NA19240 haplotype 1
2) NA19240 haplotype 1 fosmids
3) NA19238 haplotype 1

4) NA19240 haplotype 2
5) NA19240 haplotype 2 fosmids
6) NA19239 haplotype 1

NA19240 contained the GRCh37/hg19 haplotype in both its haplotype (1 and 2). Both haplotypes were validated by parental data. Fosmid sequence validated both haplotypes and parental data partially validated haplotype 1 and fully validated haplotype 2.

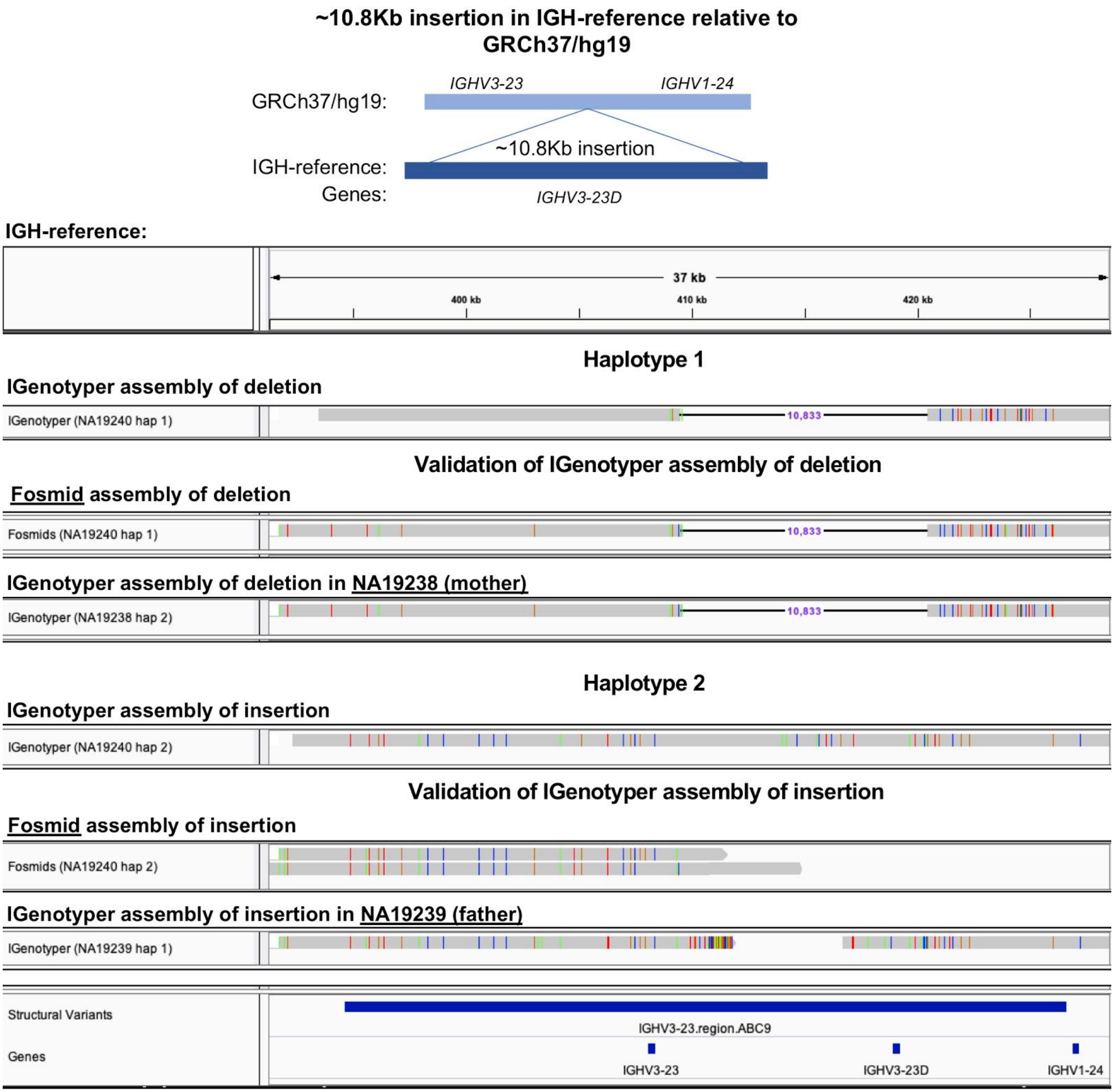

Fig. S10. Validation of duplication with *IGHV3-23D* gene in NA19240

Top: Schematic showing the duplication identified by Watson et al 2013 in NA18956 and NA12156 relative to GRCh37/hg19. The duplication was inserted in the IGH-reference.

Bottom: IGenotyper assemblies and fosmids shown in order:

1) NA19240 haplotype 1
2) NA19240 haplotype 1 fosmids

3) NA19238 haplotype 2

4) NA19240 haplotype 2

5) NA19240 haplotype 2 fosmids

6) NA19239 haplotype 1

NA19240 contained the insertion in one haplotype (2). The absence of the duplication in the alternate haplotype was validated by fosmids and parental data. The presence of the duplication was partially validated by fosmids and parental data.

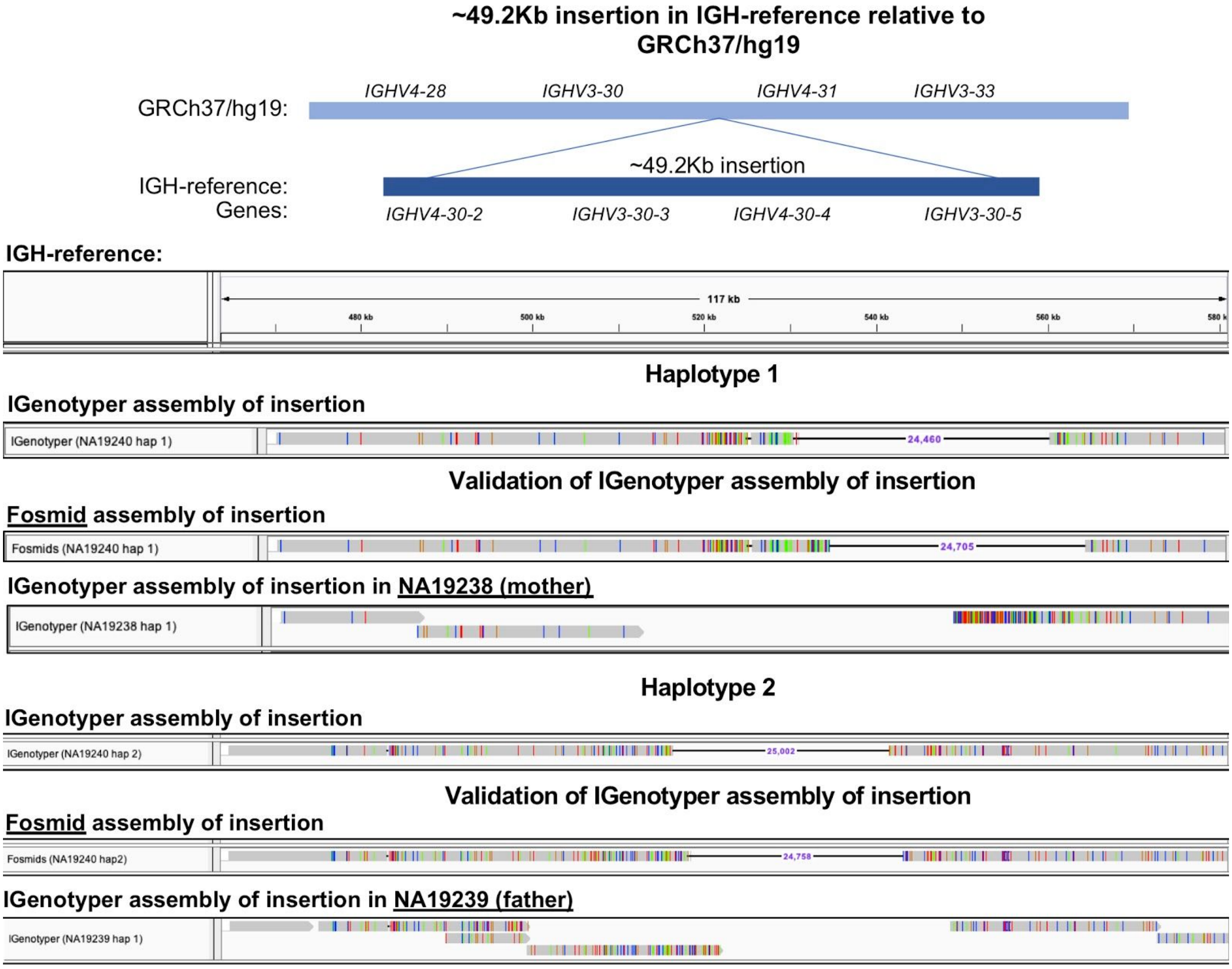

Fig. S11. Validation of previously detected duplications harboring *IGHV4-28, IGHV3-30, IGHV4-30-2, IGHV3-30-3, IGHV4-30-5, IGHV3-30-5, IGHV4-31, IGHV3-33* and *IGHV4-34* genes

Top: Schematic showing the duplication identified by Watson et al 2013 in NA18555 relative to GRCh37/hg19. The duplication was inserted in the IGH-reference.

Bottom: IGenotyper assemblies and fosmids shown in order:

1) NA19240 haplotype 1
2) NA19240 haplotype 1 fosmids
3) NA19238 haplotype 2
4) NA19240 haplotype 2
5) NA19240 haplotype 2 fosmids
6) NA19239 haplotype 1

NA19240 contained a partial insertion in haplotype 1 and 2. The partial insertions were validated by fosmids and partially by parental data. The IGenotyper assembly of haplotype 1 (1) and the fosmids of haplotype 1 (2) have a high sequence concordance (>99.99%) but due to small gaps and small number of base mismatches BLASR (the mapping aligner) shift the ~24 Kbp deletion (Table S16).

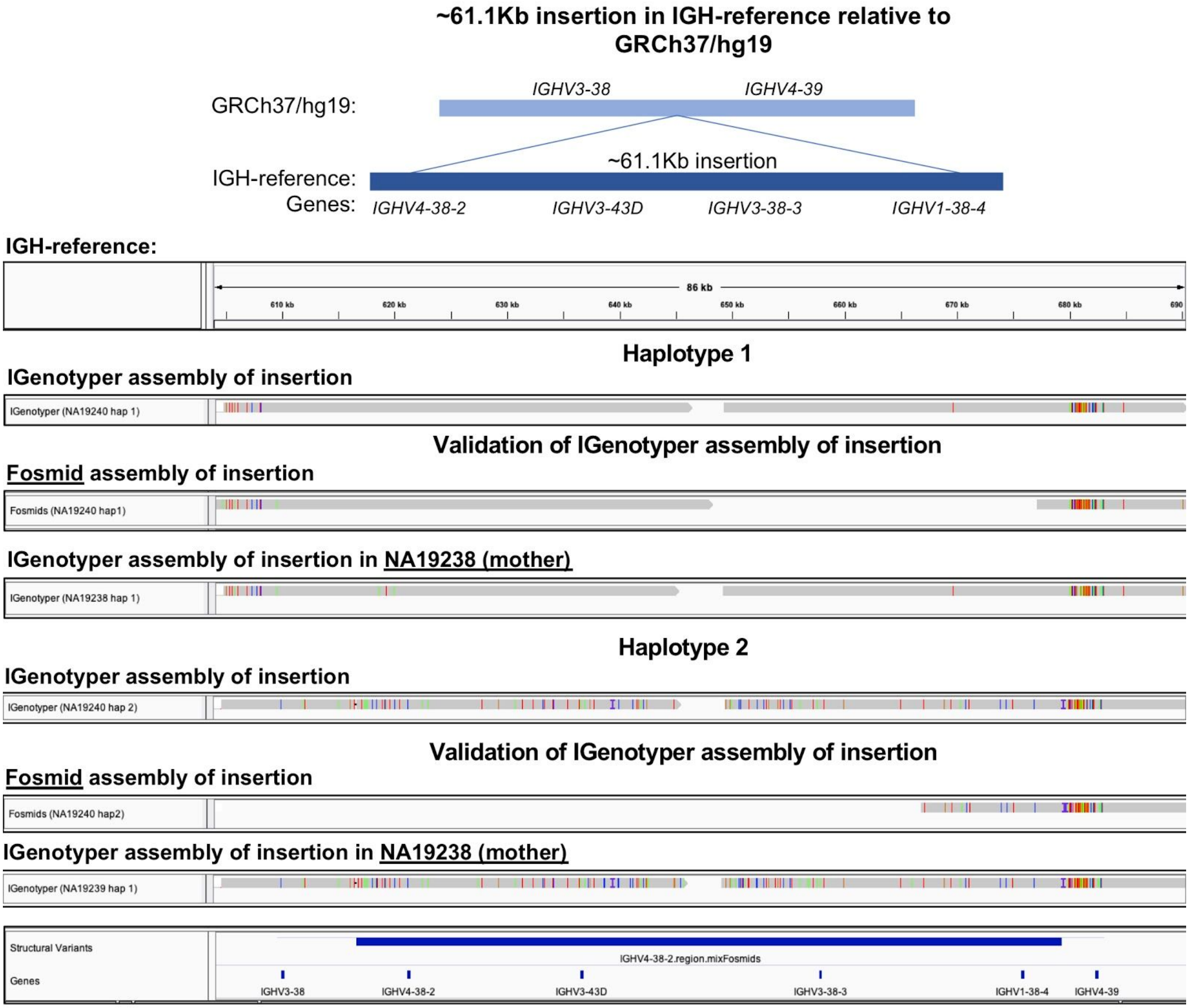

Fig. S12. Validation of insertion harboring *IGHV4-38-23, IGHV3-43D, IGHV3-38-3* and *IGHV1-38-4* gene.

Top: Schematic showing the duplication identified by Watson et al 2013 in NA15510 and NA19240 relative to GRCh37/hg19. The duplication was inserted in the IGH-reference.

Bottom: IGenotyper assemblies and fosmids shown in order:

1) NA19240 haplotype 1
2) NA19240 haplotype 1 fosmids
3) NA19238 haplotype 2

4) NA19240 haplotype 2

5) NA19240 haplotype 2 fosmids

6) NA19239 haplotype 1

NA19240 contained the insertion in both haplotypes. Both insertions were validated with parental data and partially validated by fosmids.

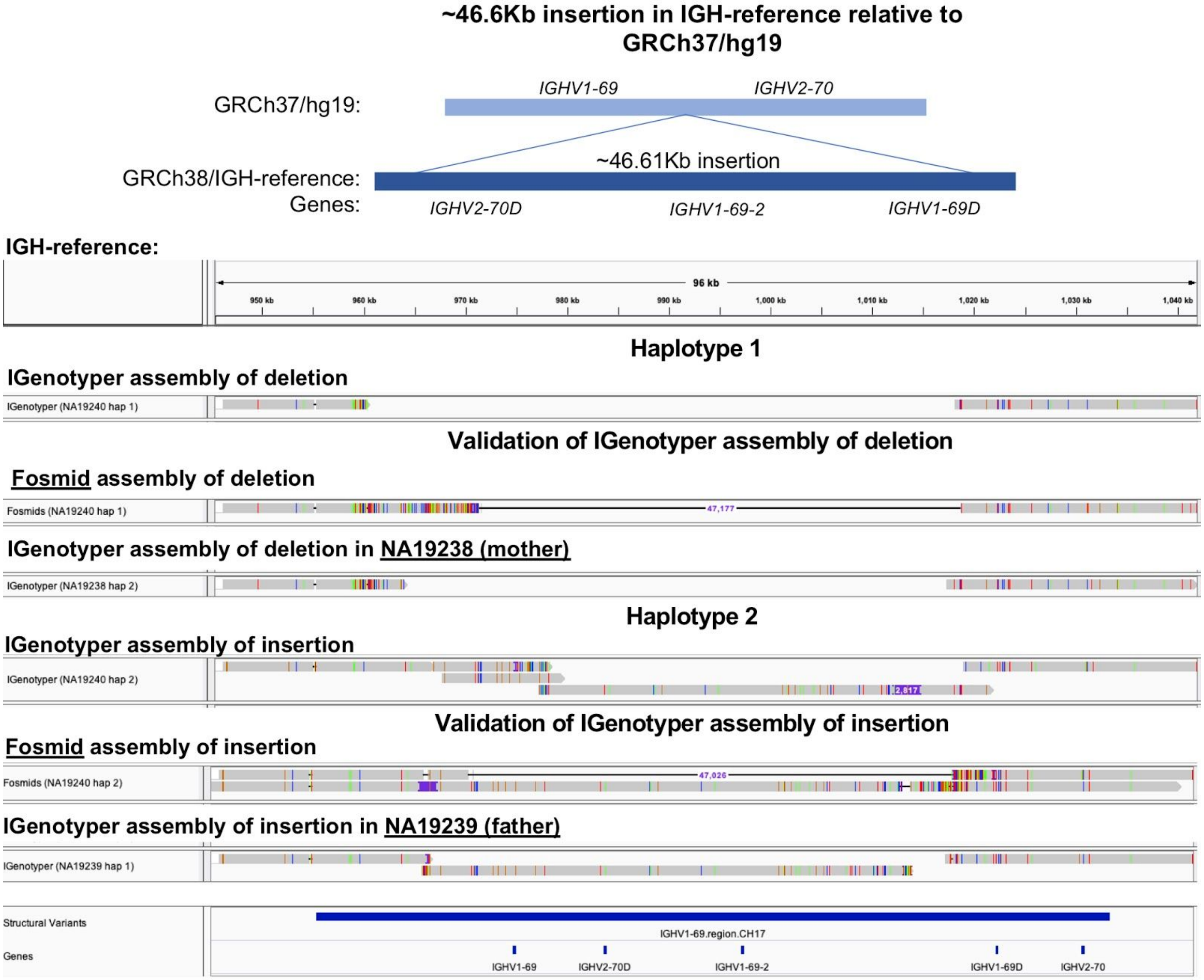

Fig. S13. Validation of previously detected insertion harboring *IGHV1-69, IGHV2-70D, IGHV1-69-2, IGH1-69D* and *IGHV2-70* genes.

Top: Schematic showing the insertion identified by Watson et al 2013 in CHM1 (GRCh38) relative to GRCh37/hg19. The insertion is present in the IGH-reference.

Bottom: IGenotyper assemblies and fosmids shown in order:

1) NA19240 haplotype 1
2) NA19240 haplotype 1 fosmids
3) NA19238 haplotype 2
4) NA19240 haplotype 2
5) NA19240 haplotype 2 fosmids
6) NA19239 haplotype 1

The insertion was present only in a single haplotype (2). The absence of the insertion was validated by fosmids and parental data. The insertion was validated by fosmids and parental data. There is evidence both in the CCS from the target-enrichment/IGenotyper sequencing run and fosmid clones that the insertion haplotype underwent a somatic mutation. The insertion haplotype passed on by the father (NA19239) was assembled as an insertion and deletion with the fosmids.

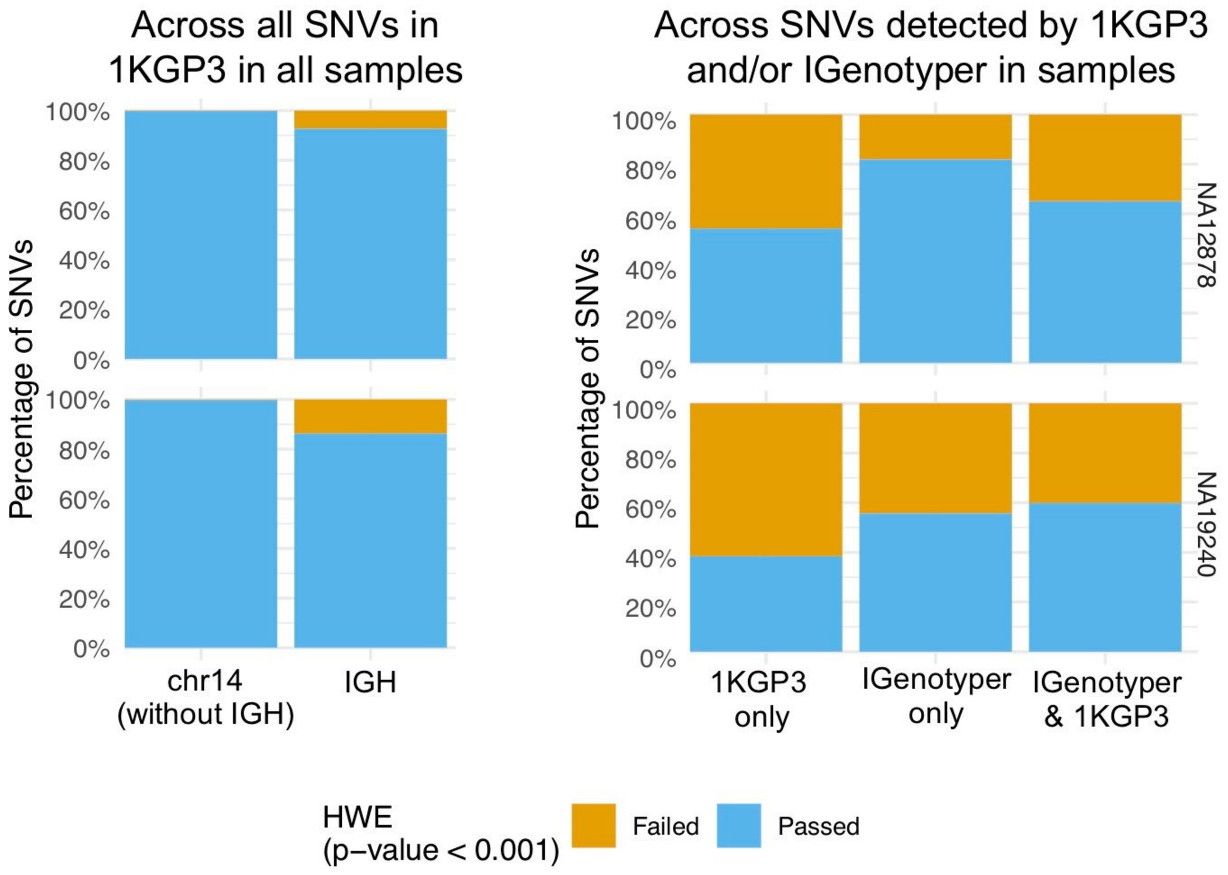

Fig. S14. Analysis of SNVs in IGH that fail or pass HWE

As a reference point, the percentage of SNVs in the 1KGP3 dataset for European samples (top left) and African samples (bottom left) in chromosome 14, excluding SNVs in the IGH locus, that fail or pass HWE was calculated. The percentage of SNVs in IGH for the same samples across all the SNVs in the 1KGP3 dataset that failed or passed HWE was also calculated. The SNVs were subsetted to those that:

1. Were found only in the 1KGP3 dataset for NA12878 and NA19240
2. Were found only by IGenotyper
3. Were found both by IGenotyper and in the 1KGP3

The percentage of SNVs that failed or passed HWE for each subset was calculated across all the African or European samples in the 1KGP3 dataset.

Table S1. Sequences used to make custom IGH reference.

IGH reference			Source sequence			
**chrom**	**start**	**end**	**chrom**	**start**	**end**	**source**
igh	1	394,082	chr14	105,860,500	106,254,581	GRCh38
igh	394,082	412,363	AC206018.3	21,438	39,718	ABC9-43993300H10
igh	412,363	427,266	AC244473.3	2,961	17,864	ABC9-43849600N9
igh	427,266	484,858	chr14	106,276,923	106,317,171	GRCh38
igh	484,858	519,568	AC231260.2	3,653	38,382	ABC11-47150400I4
igh	519,568	548,278	AC244456.2	11,919	40,628	ABC11-47354200D2
igh	548,278	562,484	KC162925.1	19,397	33,602	ABC11-49598600E10
igh	562,484	609,357	chr14	106,363,211	106,403,456	GRCh38
igh	609,357	632,150	KC162926.1	19,561	42,354	ABC10-44084700I10
igh	632,150	662,842	AC233755.2	13,040	43,732	ABC10-44145400L1
igh	662,842	685,205	AC241995.3	7,463	29,824	WI2-1707G1
igh	685,206	1,144,129	chr14	106,424,795	106,883,718	GRCh38
igh	1,144,130	1,149,130	NA	NA	NA	Gap sequence: "N"
igh	1,149,131	1,193,129	chr14	106,527,905	106,571,904	hg19

Table S4. Samples used in this study sequenced with different platforms and panels.

Sample	Replicate	Genomic DNA Source	Capture design	Approximate Insert size (kb)	PacBio Platform	# of SMRT cells	Multiplexed	Population/ Ethnicity Information
CHM1	No	Hydatidiform Mole Cell Line	A	6	RSII	2	No	Caucasian
CHM1	No	Hydatidiform Mole Cell Line	B	6	Sequel	1	No	Caucasian
CHM1	No	Hydatidiform Mole Cell Line	A+B	6	.	.	.	Caucasian
NA19238	No	Lymphoblastoid Cell Line	C	7.5	Sequel 1	1	Yes (5 samples)	YRI
NA19239	No	Lymphoblastoid Cell Line	C	7.5	Sequel 1	1	Yes (5 samples)	YRI

NA19240	No	Lymphoblastoid Cell Line	B	7.5	RSII	2	No	YRI
NA12891	No	Lymphoblastoid Cell Line	C	7.5	Sequel 1	1	Yes (5 samples)	CEU
NA12892	No	Lymphoblastoid Cell Line	C	7.5	Sequel 1	1	Yes (5 samples)	CEU
NA12878	No	Lymphoblastoid Cell Line	C	7.5	Sequel 1	1	Yes (5 samples)	CEU
NA12878	Yes	Lymphoblastoid Cell Line	B	7.5	Sequel 1	1	Yes (8 samples)	CEU
NA12878	Yes	Lymphoblastoid Cell Line	B	7.5	Sequel 1	1	Yes (8 samples)	CEU
NA12878	Yes	Lymphoblastoid Cell Line	B	7.5	Sequel 1	1	Yes (8 samples)	CEU
NA12878	Yes	Lymphoblastoid Cell Line	B	7.5	Sequel 1	1	Yes (8 samples)	CEU
NA12878	Yes	Lymphoblastoid Cell Line	B	7.5	Sequel 1	1	Yes (8 samples)	CEU
NA12878	Yes	Lymphoblastoid Cell Line	B	7.5	Sequel 1	1	Yes (8 samples)	CEU
NA12878	Yes	Lymphoblastoid Cell Line	B	7.5	Sequel 1	1	Yes (8 samples)	CEU
NA12878	Yes	Lymphoblastoid Cell Line	B	7.5	Sequel 1	1	Yes (8 samples)	CEU
Sample A (Parks et al)	No	Peripheral Blood Mononuclear Cells	A	6	RSII	2	No	Fijian

Table S5. Number and total bases of errors from incorrectly inserted sequence and missing sequence (indel errors) in the assembly of CHM1, NA19240 and NA12878.

**Sample**	**Assembly size (bp)**	**Indel errors**	**Total missing sequence (bp)**	**Total inserted sequence (bp)**	**Percentage of missing sequence**	**Percentage of inserted sequence**
CHM1	1,009,792	220	132	2,840	0.01%	0.28%
NA19240 (diploid)	1,829,616	276	217	3,984	0.01%	0.22%
NA12878 (diploid)	1,442,310	188	123	626	0.01%	0.04%

Table S6. Indel errors in the assemblies separated by size with homopolymer annotation.

Sample	Gaps greater than 100bp				Gaps between 2bp and 100bp				1bp or 2bp gaps				
	Del	Del bases (bp)	Ins	Ins bases (bp)	Del	Del bases (bp)	Ins	Ins bases (bp)	Del	Del bases (bp)	Ins	Ins bases (bp)	Del/ins in homopolymers
CHM1	0	0	3	2,521	2	6	16	222	113	126	86	97	123
NA19240	0	0	6	3,803	1	4	14	113	192	213	62	68	181
NA12878	0	0	1	520	3	10	4	16	105	113	75	90	114

Table S7. Coordinates of V(D)J recombination in the two trios whose genomic DNA were derived from LCLs.

Sample	Chrom	Start	End	V(D)J gene	IGHV genes from single haplotype	Amount of IGHV region lost in one haplotype starting from IGHV6-1 (bp)	Amount of IGHV region from both haplotypes (bp)	Percentage of IGHV region from both haplotypes
NA19240	igh	1	177,702	*IGHV2-5*	*IGHV6-1, IGHV1-2, IGHV1-3, IGHV4-4, IGHV7-4-1*	98,447	1,016,427	91.17%
NA19238	NA	NA	NA	None	None	0	1,114,874	100.00%
NA19239	igh	1	126,377	*IGHV1-2*	*IGHV6-1, IGHV1-2*	47,122	1,067,752	95.77%
NA12878	igh	1	374,857	*IGHV3-21*	*IGHV6-1, IGHV1-2, IGHV1-3, IGHV4-4, IGHV7-4-1, IGHV2-5, IGHV3-11, IGHV3-13, IGHV3-15, IGHV3-16,*	295,602	819,272	73.49%

					IGHV1-18, IGHV3-20, IGHV1-8, IGHV3-9			
NA12891	igh	1	79,558	*IGHV6-1*	*IGHV6-1*	303	1,114,571	99.97%
NA12892	igh	1	269,332	*IGHV3-13*	*IGHV6-1, IGHV1-2, IGHV1-3, IGHV4-4, IGHV7-4-1, IGHV2-5, IGHV3-11, IGHV3-13*	190,077	924,797	82.95%

Table S8. Number of haplotype blocks and heterozygous blocks

Sample	Haplotype blocks	# of heterozygous blocks	IGH reference bases in heterozygous blocks (bp)
NA19240	41	20	826,548
NA12878	49	24	424,834

Table S9. Number of fosmids used to validate assemblies.

Sample	Sequencing technology	Number of fosmids	Bases in assembly covered by fosmids	% of assembly covered by fosmid
NA19240	PacBio	85	1,505,709	82.30%
NA19240	Sanger	7	210,438	11.50%
NA12878	PacBio	73	1,180,299	81.83%
NA12878	Sanger	2	70,208	4.87%

Table S10. Mendelian inconsistencies rate in homozygous blocks

Sample	Mendelian inconsistencies	Bases from homozygous blocks	Mendelian inconsistencies rate
NA19240	27	57,313	0.047%
NA12878	23	139,029	0.017%

Table S11. Embedded structural variants in the IG-reference.

SV ID	chrom	start	end	Length	Embedded structural variant	IGHV genes

SV_1	igh	157,688	167,669	9,981	Insertion	V7-4-1
SV_2A*	igh	210,158	257,471	47,313	Complex event	V3-64D, V5-10-1
SV_3	igh	394,658	426,627	31,969	Duplication	V3-23, V3-23D
SV_4**	igh	484,922	559,858	74,936	Duplication	V4-28,V3-30,V4-30-2,V3-30-3,V4-30-4,V3-30-5,V4-31,V3-33,V4-34
SV_5	igh	609,413	682,906	73,493	Insertion	V3-38,V4-38-2,V3-43D,V3-38-3,V4-39,V1-38-4
SV_6	igh	955,770	1,033,787	78,017	Insertion	V1-69,V2-70D,V1-69-2,V2-69D,V2-70
SV_2B*	igh	114,9129	1,194,129	45,000	Complex event	V1-8,V3-9

*These complex events represent the same structural variant; on any given chromosome either V1-8/3-9 or V3-64/5-10-1 will be present.
**Given the complexity of this region, and known structural haplotype hypervariability, explicit SV genotype calls are not provided by IGenotyper for this SV.

Table S12. Alleles for IGHV genes for NA19240 and the inherited alleles in the parents of NA19240 (NA19238 and NA19239)

Gene	Haplotype 1	Haplotype 2	NA19238	NA19239
IGHV6-1	Allele lost by VDJ	*01	Allele not needed	*01
IGHV1-2	Allele lost by VDJ	*02	Allele not needed	*02
IGHV1-3	Allele lost by VDJ	*03[a]	Allele not needed	*03[a]
IGHV4-4	Allele lost by VDJ	*08	Allele not needed	*08
IGHV7-4-1	Allele lost by VDJ	*02	Allele not needed	*02
IGHV2-5	Allele lost by VDJ	*02	Allele not needed	*02
IGHV3-7	*01	*01	*01	*01
IGHV3-11	*01	*04	*01	*04
IGHV3-13	*01	*03	*01	*03
IGHV3-15	*01	*01	*01	*01
IGHV3-16	*02	*02	*02	*02
IGHV1-18	*01	*01	*01	*01
IGHV3-20	*01	*04[a]	*01	*04[a]
IGHV3-21	*01	*03	*01	*03
IGHV3-23	*01	*04	*01	*04
IGHV3-23D	Deleted	*01	Deleted	*01
IGHV1-24	*01	*01	*01	*01

IGHV2-26	*01	*01	*01	*01
IGHV4-28	*01	*07	*01	*07[b]
IGHV3-30	*18	*18	*18	*18
IGHV4-30-2	*01	*01	*01	*01
IGHV3-30-3	*01	*03	*01	*03
IGHV4-30-4	Novel	Deleted	Novel	Deleted
IGHV3-30-5	*01	Deleted	*01	Deleted
IGHV4-31	Deleted	*03	Deleted	*03
IGHV3-33	Deleted	*06	Deleted	*06
IGHV4-34	*01	*01	*01	*01
IGHV3-35	*01	*01	*01	*01
IGHV3-38	*03	*02	*03	*02
IGHV4-38-2	*02	*01	*02	*01
IGHV3-43D	*01	Novel	*01	Novel
IGHV3-38-3	*01	Novel	*01	Novel
IGHV1-38-4	*01	*01	*01	*01
IGHV4-39	*01	*01	*01	*01
IGHV3-43	*01	*01	*01	*01
IGHV1-45	*02	*02	*02	*02
IGHV1-46	*01	*03	*01	*03
IGHV3-48	*01	*01	*01	*01
IGHV3-49	*03	*03	*03	*03
IGHV5-51	*01	*03	*01	*03
IGHV3-53	*04	*02	*04	*02
IGHV1-58	*02	*02	*02	*02
IGHV4-59	*11[a]	*01	*11[a]	*01
IGHV4-61	*09[a]	*02	*09[a]	*02
IGHV3-64	*07[a]	*01	*07[a]	*01
IGHV3-66	*02	*03	*02	*03
IGHV1-69	*12	*14	*12	*14
IGHV2-70D	Deleted	*14	Deleted	*14
IGHV1-69-2	Deleted	*01	Deleted	*01

IGHV1-69D	Deleted	Novel (1-69*05)	Deleted	Novel (1-69*05)
IGHV2-70	Novel	*19[a]	Novel	*19[a]
IGHV3-72	*01	*01	*01	*01
IGHV3-73	*02	*02	*02	*02
IGHV3-74	*01	*01	*01	*01
IGHV7-81	Novel	*01	Novel	*01
IGHV1-8	*01	*03	*01	*03
IGHV3-9	*01	*03	*01	*03

[a]These alleles were novel alleles submitted to IMGT and were given allele identification prior to publication.

[b]These alleles were not detected in the parents due to decreased coverage but were present in the fosmids.

Table S13. Sequence for novel alleles detected in NA19240

Gene	Novel allele sequence
IGHV4-30-4	TCTCTGGCACAGTAATACACGGCCGTGTCTGCGGCAGTCACAGAGCTCAGCTTCA GGGAGAACTGGTTCTTGGACGTGTCTACTGATATGGTAACTCGACTCTTGAGGGA CGGGTTGTAGTAGGTGCTCCCACTGTAATAGATGTACCCAATCCACTCCAGGCCC TTCCCTGGGGGCTGGCGGATCCAGCTCCAGTAGTAATCACCACTGCTGATGGAGC CACCAGAGACAGTGCAGGTGAGGGACAGGGTCTGTGAAGGCTTCACCAGTCCTG GGCCCGACTCCTGCAGCTGCAGCTG
IGHV3-43D	TATCTTTTGCACAGTAATACAAGGCGGTGTCCTCAGCTCTCAGACTGTTCATTTGC AGATACAGGGAGTTTTTGCTGTTGTCTCTGGAGATGGTGAATCGACCCTTCACAG AGTCTGCATAGTATGTGCTACCACCATCCCAACTAATAAGAGAGACCCACTCCAG ACCCTTCCCCGGAGCTTGACGGACCCAGTGCATGGCATAATCATCAAAGGTGAAT CCAGAGGCTGCACAGGAGAGTCTCAGGAACCCCCCAGGCTGTACCACGACTCCC CCAGACTCCACCAGCTGCACTTC
IGHV3-38-3	TCTTTCTTACAGTAATACACAGCCGTGTCCTCAGCTCTCAGGCTGTTCATTTGAAG ATACAGCGTGTTCTTGGAATTGTCTCTGGAGATGGTGAATCTGCCCTTCCTGGAGT CTGCGTAGTATGTGCTACCACCACTAATGGATGAGACCCACTCCAGACCCTTCCC TGGAGCCTGGCGGACCCAGCTCATCTCATTGCTACTGACGGTGAATCCAGAGGCT GCACAGGAGAGTCTCAGGGACCCCCCAGGCTGTACCAAGACTCCCCGAGACTCC ACCAGCTGCACCTC
IGHV2-70	GTATCCGTGCACAGTAATACGTGGCTGTGTCCACAGGGTCCATGTTGGTCATTGT AAGGACCACCTGGTTTTTGGAGGTGTCCTTGGAGATGGTGAGCCTGGTCTTCAGA GATGTGCTGTAGTATTTATCATCATCCCAATCAATGAGTGCAAGCCACTCCAGGG CCTTCCCTGGGGGCTGACGGACCCAGCTCACACACATTCCACTAGTGCTGAGTGA GAACCCAGAGAAGGTGCAGGTCAGTGTGAGGGTCTGTGTGGGTTTCACCAGCGC AGGACCAGACTCCCTCAAGGTGACCTG

IGHV7-81	TATCTCGCACAGTAATACATGGCCATGTCCTCAGCCTTTAGGCTGCTGATCTGCATGTATGCTATGCTGGCAGAGGTGTCCATGGAGAAGACAAACCGTCCTGTGAAGCCCTGGGCATATGTTGGGTTCCCAGTGTAGGTGTTGAACCATCCCATCCACTCAAGCCCTTGTCCAGGGGCCTGTGGCACCCAATTCATACCATAGGTGGTGAAACTGTAACCAGAAGCCTTGCAGGAGACCTTCACTGAGGCCCCAGGCTGCTTCACCTCATGGCCAGACTGCACCAGCTGCACCTG
IGHV1-69D	IGHV1-69*05

Table S14. Alleles for IGHV genes for NA12878 and the inherited alleles in the parents of NA12878 (NA12892 and NA12891)

Gene	Haplotype 1	Haplotype 2	NA12892	NA12891
IGHV6-1	*01	Allele lost by VDJ	*01	Allele not needed
IGHV1-2	*04	Allele lost by VDJ	*04	Allele not needed
IGHV1-3	*01	Allele lost by VDJ	*01	Allele not needed
IGHV4-4	*02	Allele lost by VDJ	*02	Allele not needed
IGHV7-4-1	*01	Allele lost by VDJ	*01	Allele not needed
IGHV2-5	*02	Allele lost by VDJ	*02	Allele not needed
IGHV3-7	*01	Allele lost by VDJ	*01	Allele not needed
IGHV3-11	*01	Allele lost by VDJ	*01	Allele not needed
IGHV3-13	*01	Allele lost by VDJ	*01	Allele not needed
IGHV3-15	*07	Allele lost by VDJ	*07	Allele not needed
IGHV3-16	*02	Allele lost by VDJ	*02	Allele not needed
IGHV1-18	*01	Allele lost by VDJ	*01	Allele not needed
IGHV3-20	*04	Allele lost by VDJ	*04	Allele not needed

IGHV3-21	*01	Allele lost by VDJ	*01	Allele not needed
IGHV3-23	*01	*04	*01	*04
IGHV1-24	*01	*01	*01	*01
IGHV2-26	*2	*01	*2	*01
IGHV4-28	*07	*05	*07	*05
IGHV3-30-3	Deleted	*03	Deleted	*03
IGHV3-33	Novel	Deleted	Novel	Deleted
IGHV4-34	*01	*01	*01	*01
IGHV3-35	*01	Novel	*01	Novel
IGHV3-38	*02	*02	*02	*02
IGHV4-39	*01	*01	*01	*01
IGHV3-43	*01	*01	*01	*01
IGHV1-45	*02	*02	*02	*02
IGHV1-46	*04[a]	*01	*04[a]	*01
IGHV3-48	*01	*02	*01	*02
IGHV3-49	*03	*05	*03	*05
IGHV5-51	*01	*01	*01	*01
IGHV3-53	*02	*01	*02	*01
IGHV1-58	*01	*01	*01	*01
IGHV4-59	*01	*01	*01	*01
IGHV4-61	*01	*01	*01	*01
IGHV3-64	*02	*02	*02	*02
IGHV3-66	*01	*03	*01	*03
IGHV1-69	*04	*01	*04	*01
IGHV2-70	*15	*01	*15	*01
IGHV3-72	*01	*01	*01	*01
IGHV3-73	*01	*02	*01	*02
IGHV3-74	*01	*01	*01	*01
IGHV7-81	*01	*01	*01	*01
IGHV1-8	*01	Deleted	*01	Deleted
IGHV3-9	*01	Deleted	*01	Deleted

[a]These alleles were novel alleles submitted to IMGT and were given allele identification prior to publication.

Table S15. Sequence for novel alleles detected in NA12878

Gene	Novel allele sequence
IGHV3-33	TCTCTCGCACAGTAATACACAGCCGTGTCCTCGGCTCTCAGGCTGTTCATTTGCAG<br>ATACAGCGTGTTCTTGGAATTGTCTCTGGAGATGGTGAATCGGCCCTTCACGGAGT<br>CTGCATAGTATTTATTACTTCCATCATACCATATAACTGCCACCCACTCCAGCCCCT<br>TGCCTGGAGCCTGGCGGACCCAGTGCATGCCATAGCTACTGAAGGTGAATCCAGA<br>GGCTGCACAGGAGAGTCTCAGGGACCTCCCAGGCTGGACCACGCCTCCCCCAGAC<br>TCCACCAGCTGCACCTG
IGHV3-35	TTTCTCACACAGTAATACACAGCCGTGTCCTCGGCCCTCAGGCTATTCGTTTGCAG<br>ATACAGGGTGTTCCTGGAATTGTCTCTGGAGATGATGAATTGGCCCTTCACAGAGT<br>CTGCATAGTGCGTCCTACTGCCATTCCAACTAACACCCGATACCCACTCCAGCCCC<br>TTTCCTGGAGCCTGATGGACCCAGTTCATGTCACTGTTACTGAAGGTGAATCCAGA<br>GGCTGCACAGGAGAGTCTCAGGGATCCCCCAGGCTGTACCAAGCCTCCCCCAGAC<br>TCCACCAGCTGCACCTC

Table S16. Validation of genotyped structural variants with fosmids and parental assemblies.

SV ID	Haplotype	Percent resolved*	IGHV genes	Fosmid validation	Parent validation
SV_1	1	.	.	.	.
	2	100.00%	V7-4-1	100.00%	100.00%
SV_2B	1	100.00%	V1-8, V3-9	99.96%	100.00%
	2	98.08%	V1-8, V3-9	100.00%	100.00%
SV_3	1	100.00%	V3-23	100.00%	99.95%
	2	100.00%	V3-23, V3-23D	100.00%	100.00%
SV_4	1	100.00%	V4-28, V3-30, V4-30-2, V3-30-3, V4-30-4, V3-30-5	99.99%	99.97%
	2	100.00%	V4-28, V3-30, V4-30-2, V3-30-3, V4-31, V3-33	99.97%	99.96%
SV_5	1	95.78%	V4-38-2, V3-43D, V3-38-3, V1-38-4	100.00%	99.98%
	2	94.41%	V4-38-2, V3-43D, V3-38-3, V1-38-4	100.00%	100.00%
SV_6	1	96.95%	V1-69, V2-70	99.99%	99.99%
	2	100.00%	V1-69, V2-70D, V1-69-2, V1-69D, V2-70	99.92%	99.96%

*This refers to the number of bases resolved in the IGenotyper assembly relative to the custom IGH-reference.

Table S17. Number of SNVs lifted over to GRCh37/hg19 in NA19240 and NA12878

Sample	IGenotyper SNVs in hg19	IGenotyper not lifted to hg19	1KG Phase 3 WGS SNVs count	1KGS Phase 3 Chip SNV count
NA19240	4474	703	3120	69
NA12878	2868	737	2266	55

Table S18. Number of overlapping SNVs in NA19240 and NA12878 between IGenotyper and the 1KGP phase 3 datasets

Sample	Dataset	Count			Percentage			
		Overlap	IGenotyper only	Not in IGenotyper	Overlap (fraction of dataset)	Overlap (fraction of IGenotyper)	IGenotyper only (fraction of IGenotyper)	Not in IGenotyper (fraction of dataset)
NA19240	1KG WGS	2,578	1,896	542	82.63%	57.62%	42.38%	17.37%
NA19240	1KG Chip	60	4,414	9	86.96%	1.34%	98.66%	13.04%
NA12878	1KG WGS	2,190	678	76	96.65%	76.36%	23.64%	3.35%
NA12878	1KG Chip	52	2816	3	94.55%	1.81%	98.19%	5.45%

Table S19. Number of SNVs within accessible regions defined by 1KGP

Sample	Category	Total SNVs	SNVs in pilot regions	SNVs in strict regions	SNVs in in-accessible regions with pilot criteria	SNVs in in-accessible regions with strict criteria
NA12878	IGenotyper only	678	342	20	49.56%	97.05%
NA12878	Not in IGenotyper	76	62	23	18.42%	69.74%
NA19240	IGenotyper only	1,896	1289	165	32.01%	91.30%
NA19240	Not in IGenotyper	542	487	210	10.15%	61.25%

Table S20. Number of SNVs passing or failing ($p < 0.001$) Hardy-Weinberg equilibrium

Sample	Category	Total	Fail (thres=0.001)	% Fail	% Pass

NA12878	chr14 (no IGHV locus)	2,607,046	5,793	0.22%	99.78%
	IGHV locus	34,393	2,518	7.32%	92.68%
	Overlap with IGenotyper	2,162	755	34.92%	65.08%
	IGenotyper only	127	23	18.11%	81.89%
	Not in IGenotyper	74	34	45.95%	54.05%
NA19240	chr14 (no IGHV locus)	2,607,046	7,689	0.29%	99.71%
	IGHV locus	34,393	4,740	13.78%	86.22%
	Overlap with IGenotyper	2,163	869	40.18%	59.82%
	IGenotyper only	925	410	44.32%	55.68%
	Not in IGenotyper	229	141	61.57%	38.43%

Table S21. Genotypes for NA19240 embedded structural variants in custom IGH reference.

**SV ID**	**Genotype**	**IGHV gene genotypes**	**BioNano Support**
SV_1	./0	./V7-4-1	Yes
SV_2B	0/0	V3-9,V1-8/V3-9,V1-8	No
SV_2A	1/1	./.	No
SV_3	0/1	V3-23D,V3-23/V3-23	Yes
SV_5	0/0	V4-38-2,V3-43D,V3-38-3,V1-38-4/V4-38-2,V3-43D,V3-38-3, V1-38-4	Yes
SV_6	0/1	V1-69,V2-70/V1-69,V2-70D,V1-69-2,V1-69D,V2-70	Yes

Table S22. Statistics from multiplexing replicates of NA12878

**Replicate**	**CCS coverage**	**Subread coverage**	**Sequence concordance**	**Amount of assembly recapitulated**	**Overlapping variants**	**Missed variants**	**Additional variants**
NA12878_1	72.98	660.04	99.97%	98.46%	1,706	429	45
NA12878_2	74.11	666.82	99.99%	98.57%	2,029	56	48
NA12878_3	68.64	623.72	99.99%	98.74%	2,027	58	49
NA12878_4	65.58	594.86	99.99%	98.54%	2,061	24	41
NA12878_5	95.37	819.49	99.99%	93.82%	2,052	34	41

NA12878_6	41.30	358.56	99.99%	95.74%	1,972	112	15
NA12878_7	101.62	872.87	99.98%	94.13%	2,042	44	66
NA12878_8	97.73	837.55	99.98%	94.37%	2,048	38	49
Average	77.76	681.98	99.99%	96.27%	2033	52.29	44.14
